# Different oral and gut microbial profiles in those with Alzheimer's disease consuming anti-inflammatory diets

**DOI:** 10.3389/fnut.2022.974694

**Published:** 2022-09-15

**Authors:** Lili Chen, Bixia Wang, Jinxiu Liu, Xiaoqi Wu, Xinhua Xu, Huizhen Cao, Xinli Ji, Ping Zhang, Xiuli Li, Zhaoyi Hou, Hong Li

**Affiliations:** ^1^Shengli Clinical Medical College of Fujian Medical University, Fuzhou, China; ^2^The School of Nursing, Fujian Medical University, Fuzhou, China; ^3^Fujian Provincial Hospital, Fuzhou, China; ^4^Fujian University of Traditional Chinese Medicine, Fuzhou, China

**Keywords:** oral microbial, gut microbial, dietary inflammatory index, Alzheimer's disease, systemic inflammation

## Abstract

The number of people living with Alzheimer's disease (AD) is increasing alongside with aging of the population. Systemic chronic inflammation and microbial imbalance may play an important role in the pathogenesis of AD. Inflammatory diets regulate both the host microbiomes and inflammatory status. This study aimed to explore the impact of inflammatory diets on oral-gut microbes in patients with AD and the relationship between microbes and markers of systemic inflammation. The dietary inflammatory properties and the oral and gut microorganisms were analyzed using the dietary inflammatory index (DII) and 16S RNA in 60 patients with AD. The α-diversity was not related to the DII (*p* > 0.05), whereas the β-diversity was different in the oral microbiomes (R^2^ = 0.061, *p* = 0.013). In the most anti-inflammatory diet group, *Prevotella* and *Olsenella* were more abundant in oral microbiomes and *Alistipes, Ruminococcus, Odoribacter*, and unclassified *Firmicutes* were in the gut microbiomes (*p* < 0.05). Specific oral and gut genera were associated with interleukin-6 (IL)-6, complement 3 (C3), high-sensitivity C-reactive protein (hs-CRP), IL-1β, IL-4, IL-10, IL-12, and tumor necrosis factor-α (TNF-α) (*p* < 0.05). In conclusion, anti-inflammatory diets seem to be associated with increased abundance of beneficial microbes, and specific oral and gut microbial composition was associated with inflammatory markers.

## Introduction

Alzheimer's disease (AD), the most common cause of dementia, is an age-related neurodegenerative disease with an insidious onset that manifests primarily as progressive cognitive impairment, most often in people over 65 years of age (1). With the aging process, more and more older adults are at risk of developing AD. The predicted number of those with AD worldwide is reported to be 66 million in 2030 and 115 million in 2050 (2), and China will have 8–11 million patients with AD by 2050 (3). Given the high incidence and the absence of effective pharmacological treatments for AD, experts emphasize identifying risk factors for early prevention and intervention (4). Diet is considered an important means of prevention and intervention (5).

Aging is accompanied by a chronic, progressive pro-inflammatory response called inflammaging. Inflammaging gradually disrupts the balance of microbiota, resulting in decreased microbial diversity and increased harmful microorganisms (6, 7). Multiple studies have found that elevated serum C-reactive protein (CRP), interleukin-6 (IL-6), and tumor necrosis factor-α (TNF-α) levels were found in those with AD (8–10), which is associated with neurological and peripheral inflammation (11, 12). Recent studies suggested a significant reduction in microbial diversity and alteration of gut microbial composition in patients with AD (13, 14). These changes had a significant connection to the diet (15–18), especially diets with inflammatory components (19, 20). A Western diet—characterized by high sugar and fat—is associated with chronic low-level systemic inflammation and reduced gut microbial diversity (21, 22); altered microbiota have been implicated in AD development (23, 24). However, a Mediterranean diet based on fish, grains, vegetables, fruits, nuts, and olive oil not only increases the diversity of the intestinal microflora (25) and reduces harmful flora such as those of the *Aspergillus*, but it also reduces the level of inflammation in the body and reduces the risk of AD (26). This suggests that the food consumed by an individual has an inflammatory potential and that it affects the microbial community. Inflammatory dietary components can promote the growth of certain microorganisms, causing changes in, for example, intestinal permeability and metabolic function, and leading to inflammation (27). Therefore, the dietary inflammatory index (DII) was created to measure the potential for the diet to be inflammatory through comprehensively assessing the relationship between diet and markers of inflammation (CRP, interleukin (IL)-1β, IL-4, IL-6, IL-10, and TNF-α (28, 29). A study shows that participants with higher DII had lower microflora diversity and a positive correlation with CRP≥3 mg/L, suggesting that a pro-inflammatory diet reduces microbial diversity and increases the level of inflammation in the host (30). In addition, food provides substrates for microflora, which subsequently produce metabolites that modulate inflammation (31, 32). For example, the Mediterranean diet increases the production of short-chain fatty acids (SCFAs) (33, 34). Refined processed foods, saturated fats, trans fats and sugars, and a low intake of fruits and vegetables have been shown to alter the gut microbiota and the function of the gastrointestinal tract, reducing the formation of SCFAs and increasing inflammation in the host (35).

These findings suggest that the food consumed affects an individual's microbial communities and has the potential to be inflammatory. In addition to intestinal microbes, oral microecological are associated with AD pathogenesis. A study shows that the richness and diversity of saliva microbiota detected in AD patients are lower than healthy controls (36). Other studies have reported detecting more oral bacteria in the brains of AD patients (37, 38). Further oral microecological dysregulation and intestinal inflammation are directly associated with intestinal barrier dysfunction and increased intestinal permeability and may cause malignant destruction of neurons (39–43). Harding et al. advocate that maintaining oral and intestinal health greatly benefits the host (44). Despite the proposed role of diet-related inflammation and oral-gut microbiota in the development or progression of AD, there have been no comprehensive surveys of the diet about inflammation and oral-gut microbiome in individuals with AD. Given that an inflammation-associated diet may interact with microorganisms to regulate organismal inflammation. Therefore, the aim of this study was to investigate the effect of an inflammation-related diet on oral-gut microbial diversity and composition. In addition to initially exploring the relationship between microbial composition and markers of inflammation.

## Materials and methods

### Participants

A total of 60 subjects with AD aged between 60 and 80 years and community-dwelling Han residents were recruited from the Memory Clinic of Fujian Provincial Hospital (Fujian, China) between February 2020 and December 2021. The participants with AD in this study were diagnosed according to the 2018 National Institute of Aging and Alzheimer's Association (NIA-AA) guidelines and the Diagnostic and Statistical Manual of Mental Disorders (fifth edition) by experienced neurologists (45). And the patients with AD were divided into mild, moderate, and severe AD groups according to the mini-mental state examination (MMSE) scores (mild: 21–26, moderate: 11–20, and severe: 0–10) (46). The exclusion criteria included: (1) other causes of dementia or other types of dementia; (2) a family history of dementia; (3) any kind of neurodegenerative disease, such as Parkinson's disease; (4) confirmed mental illness, such as schizophrenia; (5) severe cardiac, pulmonary, hepatic, or renal disease, or any tumor; (6) intestinal diseases, such as irritable bowel syndrome; (7) taking antibiotics, glucocorticoids, or probiotics within 1 month; (8) known active infections, such as viral, bacterial, or fungal infections, or other autoimmune diseases; (9) infected with a SARS-CoV-2; (10) with severe auditory, visual, or motor deficits that may interfere with cognitive testing were also excluded. In addition, the oral health status of the participants with AD was measured to exclude subjects with the following conditions in the 2 months before the study: oral or dental surgery, inflammation of the oral or perioral tissues, or other chronic diseases of the oral cavity.

The study was registered at the Chinese Clinical Trial Registry (ChiCTR2100041749) and approved by the Ethics Committee of Fujian Provincial Hospital (ref no. K2020-09-025). All participants provided written informed consent before participation.

### Dietary data collection

A validated Chinese annual semi-quantitative food frequency questionnaire (FFQ), which has good reliability and validity and contains 17 food groups with a total of 149 food items, was repeated six times over one-year to collect 24-h recalls for 3 days from each subject every 2 months to elucidate daily variation and seasonality of food changes, as well as medium-term changes in dietary habits during the study period. In our study, FFQ was used to assess subjects' dietary intake data for the past year (47). Subjects quantified their food intake by referring to the Retrospective Dietary Survey Supplementary Reference Physical Atlas, which provided photographs of food items in different portion sizes. For example, consumption of edible oil and condiments by those in the household was measured monthly. Participants with >20% of missing FFQ items and abnormal total energy intake were excluded from this study to avoid outlier effects, i.e., an overall intake for males <800 or >4,000 kcal/day and for females <500 or >3,500 kcal/day (48). It is worth mentioning that each AD patient had a relative or guardian present at the time of the survey to confirm the patient's dietary habits. And when patients were unable to fill out the questionnaire on their own, we completed it by consulting their family members, with priority given to partners and co-residents, to confirm the authenticity of the questionnaire content.

### Dietary inflammatory index

The DII is a literature-based tool that computes the inflammatory properties of a diet, based on the association of certain food and dietary constituents with defined inflammatory hallmarks: CRP, TNF-α, IL-1β, IL-4, IL-6, and IL-10 (28). In this study, based on the actual food parameters obtained from the FFQ, 24 food parameters were ultimately included to calculate the DII. Pro-inflammatory ingredients include carbohydrates, energy, fat, protein, saturated fat, cholesterol, and iron, and anti-inflammatory ingredients include carotenoids, caffeine, fiber, monounsaturated fatty acids, polyunsaturated fatty acids, riboflavin, green tea, onions, garlic, ginger, vitamin A, vitamin C, vitamin E, magnesium, zinc, selenium, thiamin (Table 1) (28). Components such as eugenol, saffron, isoflavones, pepper, thyme/oregano, and rosemary were excluded because of a lack of related information in the FFQ recordings.

**Table 1:** Pro-inflammatory and anti-inflammatory ingredients.

**Anti-inflammatory ingredients**	**Pro-inflammatory ingredients**
Carotenoids (mg/day)	Carbohydrates (g/day)
Caffeine (g/day)	Energy (Kcal/day)
Fiber (g/day)	Fat (g/day)
MUFA (g/day)	Protein (g/day)
PUFA (g/day)	SFA (g/day)
Riboflavin (mg/day)	Cholesterol (mg/day)
Green tea (g/day)	Iron (mg/day)
Onions (g/day)	-
Garlic (g/day)	-
Ginger (g/day)	-
Vitamin A (RE/day)	-
Vitamin C (mg/day)	-
Vitamin E(mg/day)	-
Magnesium (mg/day)	-
Zinc (mg/day)	-
Selenium(mg/day)	-
Thiamin (mg/day)	-

To avoid randomness caused by the use of individual initial intake values, the actual intake of each food parameter was normalized to a z-score based on the global average intake and standard deviation for 11 countries. Individual z-scores were then converted to centered percentiles. Each centered percentile was multiplied by the standardized overall inflammatory effect score. Finally, the DII scores for all food parameters were summed to obtain the individual DII scores. The final score is a continuous measure, interpreted as strongly anti-inflammatory (the lowest score) to strongly pro-inflammatory (the highest score).

### The international physical activity questionnaire

Physical activity (PA) in the most recent week was assessed using the short form of the International Physical Activity Questionnaire (49). The questionnaire asked whether subjects had performed any activities from the following categories during the previous week: walking; moderate activity (household activity or child care); and vigorous activity (running, swimming, or other sports activities). Metabolic equivalent (MET) hours per week were calculated using corresponding MET coefficients (3.3, 4.0 and 8.0, respectively) according to the following formula: MET coefficient of activity × duration (hours) × frequency (days). Total PA levels were assessed by combining the scores for different activities.

### Sample collection and handling

Participants were instructed not to brush their teeth on the morning of the sampling day and the night before. After a trained dentist scratched the supragingival debris with sterilized cotton balls, a subgingival plaque was collected with 40# sterilized paper points (Gapadent, Tianjin, China) that were gently inserted into the deep periodontal pocket for 20 s. Once removed from the periodontal pocket, the paper point was placed into a 1-mL sterile cryopreservation tube (50). No sample was collected if a patient had no teeth or dental implants. All subgingival plaque samples were stored at −80°C before processing.

Each participant was asked to collect a fresh fecal sample in the morning. Because several community-dwelling older subjects could not send their samples to the hospital immediately, they were given fecal collection containers (SARSTEDT, Germany) with approximately 5 mL of special cytoprotective agents to preserve the DNA in the stool at an approximate temperature for 10–14 days. The fecal samples were then transferred to the laboratory and stored at −80°C before processing.

Blood samples were collected from the participants after an overnight fast by trained laboratory staff. Immediately after the sample of venous blood was collected, it was centrifuged and stored at −80°C before processing. The serum concentrations of IL-6, complement 3 (C3), high-sensitivity (hs-CRP), IL-1β, IL-4, IL-10, IL-12, as well as TNF-α, were determined with an enzyme-linked immunosorbent assay method according to the manufacturer's directions (Elabscience, Wuhan, China). Oral and blood samples were collected on the same day, except for fecal sample.

### DNA extraction and 16S rRNA gene amplicon sequencing

DNA extraction and 16S rRNA gene amplicon sequencing DNA extraction, PCR amplification, and the sequencing of the V3–V4 hypervariable regions of the bacterial 16S rRNA gene based on all oral and gut DNA samples were undertaken at the DNA Sequencing and Genomics Laboratory of Sangon BioTech (Shanghai). Following the manufacturer's instructions, total community genomic DNA extraction was performed using an E.Z.N.A. Soil DNA Kit (Omega, USA). PCR was started immediately after the DNA was extracted. The 16S rRNA V3–V4 amplicon was amplified using KAPA HiFi Hot Start Ready Mix (2×) (TaKaRa Bio Inc., Japan). Two polyacrylamide gel electrophoresis-purified universal bacterial 16S rRNA gene amplicon PCR primers were used: the amplicon PCR forward primer (5′-CCTACGGGNGGCWGCAG-3′) and the amplicon PCR reverse primer (5′-GACTACHVGGGTATCTAATCC-3′). PCR was performed using a thermal cycler (Applied Biosystems 9700, USA) using the following program: one cycle of denaturing at 95°C for 3 min; five cycles of denaturing at 95°C for 30 s, annealing at 45°C for 30 s, and elongation at 72°C for 30 s; 20 cycles of denaturing at 95°C for 30 s, annealing at 55°C for 30 s, and elongation at 72°C for 30s; and a final extension at 72°C for 5 min. The PCR products were checked through a separation with electrophoresis in 1% (w/v) agarose gels in Tris, boric acid, and EDTA (TBE) buffer, staining with ethidium bromide, and visualizing under ultraviolet light.

Sequencing was then performed using the Illumina MiSeq system (Illumina MiSeq, California, USA). The raw sequencing reads were detected using FastQC software to remove the primer region and low-quality sequences. The chimera sequences arising from the PCR amplification were detected and excluded using Mothur (http://www.mothur.org) based on the GreenGenes database. The high-quality reads that reached a 97% nucleotide similarity were clustered into operational taxonomic units (OTUs) according to the Ribosomal Database Project database. Summaries of the taxonomic distributions of OTUs were constructed using these taxonomies and were used to calculate the relative abundance of microbiota at the phylum and genus taxonomic levels.

### Bioinformatic analysis

The α- (Shannon, Simpson, Chao1, and ACE index) and β-diversity analyses (Bray–Curtis dissimilarity and principal coordinate analysis [PCoA]) were conducted using QIIME and R software to compare the similarity among samples in terms of the diversity of species. Analysis of variance (ANOVA) or the Kruskal–Wallis test was performed to evaluate α-diversity among the different groups. Permutational multivariate analysis of variance (PERMANOVA) was employed to identify the different microbial communities among groups. The relative abundance diagram of flora species was mainly used to visualize the results of species annotation. STAMP analysis was used to identify species that differed in abundance between two or more groups. The key taxa responsible for the differences in the oral and gut microbiota between AD groups were identified using the linear discriminant analysis effect size (LEfSe) for biomarker discovery (51). Spearman correlation coefficients were used to detect relationships between the taxa and inflammatory markers. A significant α of 0.05 and an effect size threshold of 2 were used for all biomarkers discussed in this study.

### Statistical analysis

Baseline characteristics of participants are presented as means ± SD, medians (interquartile ranges [IQRs]), and numbers (percentages). Comparisons of continuous variables between groups were made using ANOVA or rank sum tests, and group differences were compared using a chi-squared test or Fisher's exact test for categorical variables. Kruskal-Wallis test was used to analyze the skewness distribution data. Furthermore, when the distribution of variables was skewed (as for IL-6, C3, hs-CRP, IL-1β, IL-4, IL-10, IL-12, and TNF-α), the values were converted to their natural logarithm.

DII scores were ranked and split into approximately equal tertiles, with tertile 1 (T1) representing the most anti-inflammatory diet group and tertile 3 (T3) representing the most pro-inflammatory group. We performed multiple linear regression analysis to examine associations between each tertile of DII scores and inflammatory indicators, and tertile 1 (T1) was considered the reference. In Model 1, we did not adjust for covariates. In Model 2, we adjusted for age and gender. In Model 3, we adjusted for the variables in Model 2 with the addition of education, body mass index (BMI), smoking, alcohol consumption, physical activity, hypertension, diabetes, hyperlipidemia, coronary heart disease, and cerebrovascular disease. Tests for trends were performed by assigning the median value for each tertile and modeling this as a continuous variable. Energy adjustment was done using the residual approach that was explained previously by Willett et al. (52). For calculating energy-adjusted dietary intakes, each of the dietary components is regressed on their total energy intake and residual values were added to their actual mean intake to estimate energy-adjusted values. Statistical analysis was performed using SAS version 9.4 software (SAS Institute Inc., Cary, USA) and STAMP v2.1.3 (53). The R package and GraphPad Prism v6.0 were used to prepare the graphs. All tests of significance were two-sided and *p* < 0.05 was considered statistically significant.

## Results

### Baseline characteristics of DII levels and inflammatory indicators in patients with AD

The DII range was −0.021 (the most anti-inflammatory diet) to 1.38 (the most pro-inflammatory diet). According to the DII score, the tertiles were divided from low to high, T1 (tertile 1) means the most anti-inflammatory diet group, T2 (tertile 2) means no anti-inflammatory/pro-inflammatory diet group, and T3 (tertile 3) means the most pro-inflammatory diet group. The baseline characteristics of 60 patients with AD including T1 (*n* = 20), T2 (*n* = 20), and T3 (*n* = 20) are shown in Table 2. Among all the variables examined, significant differences were observed only for the DII score, mean total daily energy intake, and education level (*p* < 0.05). Patients with higher DII scores (i.e., more pro-inflammatory diets) tended to have lower total energy intakes and education levels. However, there was no statistically significant difference across DII levels in the distribution of other baseline characteristics and inflammation markers (*p* > 0.05). To further analyze whether there is an association between each tertile of DII scores and inflammatory indicators, we used multiple linear regression with model adjustment for con-founders. The results still showed that none of the inflammatory indicators was statistically significant among the groups (see Supplementary material for details).

**Table 2:** Baseline characteristics of DII levels and inflammatory indicators in patients with AD.

**Variable**	**T1 (*n* = 20)**	**T2 (*n* = 20)**	**T3 (*n* = 20)**	**F/χ2**	***p*-Value**
	**<–0.021**	**–0.021–1.308**	**≥1.308**		
DII score	−0.5 ± 0.9	1.2 ± 0.2	2.4 ± 0.6	99.502	<0.001
Age (years)	75.9 ± 11.92	73.0 ± 9.25	76.1 ± 9.11	1.481	0.558
BMI (kg/m^2^)	22.2 ± 2.48	23.2 ± 3.44	24.7 ± 3.54	5.779	0.066
Energy intake (kcal/day)	2,457.7 ± 445.46	1,931.1 ± 280.41	1,462.0 ± 399.13	34.269	<0.001
Sex, *n* (%)				3.636	0.162
Male	12 (60.0)	9 (45.0)	6 (30.0)		
Female	8 (40.0)	11 (55.0)	14 (70.0)		
Education level, n (%)				11.917	0.044
None	0 (0.0)	7 (35.0)	5 (25.0)		
Primary school	5 (25.0)	5 (25.0)	5 (25.0)		
Middle school	9 (45.0)	6 (30.0)	9 (45.0)		
High school or higher	6 (30.0)	2 (10.0)	1 (5.0)		
Income (yuan), n (%)				4.602	0.626
<3,000	4 (20.0)	3 (15.8)	5 (25.0)		
3,000–5,000	5 (25.0)	2 (10.5)	2 (10.0)		
5,000–10,000	3 (15.0)	7 (36.8)	7 (35.0)		
>10,000	8 (40.0)	7 (36.8)	6 (30.0)		
Smoking status, *n* (%)				1.543	0.821
Never	12 (63.2)	16 (80.0)	15 (75.0)		
Former	4 (21.1)	2 (10.0)	3 (15.0)		
Current	3 (15.8)	2 (10.0)	2 (10.0)		
Alcohol status, *n* (%)				5.434	0.347
Current	1 (5.0)	2 (10.0)	1 (5.0)		
Former	6 (30.0)	2 (10.0)	7 (35.0)		
Never	13 (65.0)	16 (80.0)	12 (60.0)		
Diabetes, *n* (%)				0.745	0.799
Yes	4 (20.0)	4 (20.0)	6 (30.0)		
No	16 (80.0)	16 (80.0)	14 (70.0)		
Hypertension, *n* (%)				1.833	0.400
Yes	6 (30.0)	7 (35.0)	10 (50.0)		
No	14 (70.0)	13 (65.0)	10 (50.0)		
Hyperlipidemia, *n* (%)				1.111	0.863
Yes	2 (10.0)	1 (5.0)	3 (15.0)		
No	18 (90.0)	19 (95.0)	17 (85.0)		
Coronary heart disease, *n* (%)				2.353	0.474
Yes	2 (10.0)	2 (10.0)	5 (25.0)		
No	18 (90.0)	18 (90.0)	15 (75.0)		
Cerebrovascular disease, *n* (%)				2.143	0.532
Yes	0 (0.0)	2 (10.0)	2 (10.0)		
No	20 (100.0)	18 (90.0)	18 (90.0)		
Physical activity (METs × h/wk), medians (IQR)	35.88 (17.70–49.76)	74.20 (11.55–181.30)	24.33 (11.55–67.55)	3.374	0.185
Systolic blood pressure (mmHg) medians (IQR)	120.50 (112.00–136.00)	127.00 (120.00–141.00)	128.50 (118.00–139.00)	1.593	0.511
Diastolic blood pressure (mmHg)	77.9 ± 11.57	81.8 ± 10.50	77.7 ± 8.75	4.312	0.408
Severity of AD				1.459	0.241
Mild	10 (50.0)	7 (35.0)	9 (45.0)		
Moderate	10 (50.0)	9 (45.0)	9 (45.0)		
Severe	0 (0.0)	4 (20.0)	2 (10.0)		
IL-1β (pg/mL)	3.5 ± 0.78	3.5 ± 0.68	3.6 ± 0.70	1.433	0.789
IL-4 (pg/mL)	3.2 ± 0.62	3.0 ± 0.65	3.3 ± 0.55	1.999	0.412
IL-10 (pg/mL)	5.8 ± 0.65	5.9 ± 0.84	5.7 ± 1.12	1.390	0.522
IL-12 (pg/mL)	3.2 ± 0.40	2.8 ± 1.25	2.9 ± 0.89	1.057	0.459
TNF-α (pg/mL)	4.2 ± 0.42	3.8 ± 0.57	4.0 ± 0.56	3.689	0.164
hs-CRP (mg/L)	2.1 ± 0.63	2.1 ± 0.49	1.8 ± 0.61	2.561	0.341
IL-6 (pg/mL)	2.8 ± 0.61	2.8 ± 0.52	2.8 ± 0.66	0.095	0.942
C3 (μg/mL)	5.8 ± 0.53	5.6 ± 1.00	5.8 ± 0.62	0.035	0.911

Data are presented as mean ± SD, medians (interquartile range, IQR), or numbers (%). According to the DII score, the tertiles were divided from low to high, T1 (tertile 1) means the most anti-inflammatory diet group, T2 (tertile 2) means no anti-inflammatory/pro-inflammatory diet group, and T3 (tertile 3) means the most pro-inflammatory diet group. The MMSE score was used for AD severity according to the guidelines, with 21 to 26 as mild cognitive impairment, 11 to 20 as moderate cognitive impairment, and 0 to 10 as severe cognitive impairment. METs, metabolic equivalents; BMI, body mass index; IL-1β, interleukin-1; IL-4, interleukin-4; IL-6, interleukin-6; IL-10, interleukin-10; IL-12, interleukin-12; TNF-α, tumor necrosis factor-α; C3, complement C3; hs-CRP, high-sensitivity C-reactive protein.

The energy-adjusted dietary intakes for participants in the different inflammatory diet groups are presented in Table 3. Compared to the most anti-inflammatory diet group, individuals with the most pro-inflammatory diet group consumed significantly lower amounts of energy, protein, fat, carbohydrate, fiber, vitamin A, carotenoids, thiamin, riboflavin, vitamin C, vitamin E, magnesium, iron, zinc, selenium, and PUFA (all *p* < 0.05).

**Table 3:** Food group and nutrient intake of AD patients in different inflammatory diet groups.

**Variable**	**T1 (*n* = 20)**	**T2 (*n* = 20)**	**T3 (*n* = 20)**	***p*-Value**
	**<-0.021**	**−0.021–1.308**	**≥1.308**	
**Nutrients**				
Energy (Kcal/day)	2,457.7 ± 445.46	1,931.1 ± 280.41	1,461.9 ± 399.13	<0.001
Protein (g/day)	91.0 ± 17.00	74.1 ± 15.81	52.9 ± 16.09	<0.001
Fat (g/day)	41.0 ± 12.61	33.7 ± 9.87	23.4 ± 9.93	<0.001
Carbohydrate (g/day)	428.6 ± 88.18	326.8 ± 53.9	259.9 ± 75.07	<0.001
Fiber (g/day)	19.7 ± 3.47	13.9 ± 1.59	9.4 ± 2.94	<0.001
Cholesterol (mg/day)	440.5 ± 167.86	334.6 ± 170.03	291.8 ± 137.14	0.014
Vitamin A (RAE/day)	3,466.9 ± 723.21	2,540.7 ± 627.98	1,682.8 ± 695.81	<0.001
Carotenoids (μg/day)	2,614.9 ± 601.17	1,846.1 ± 485.93	1,155.5 ± 560.73	<0.001
Thiamin (mg/day)	1.1 ± 0.24	0.9 ± 0.32	0.63 ± 0.19	<0.001
Riboflavin (mg/day)	1.2 ± 0.19	0.9 ± 0.19	0.7 ± 0.24	<0.001
Vitamin C (mg/day)	100.8 ± 31.92	61.9 ± 19.59	43.9 ± 19.97	<0.001
Vitamin E (mg/day)	23.6 ± 8.72	16.3 ± 7.55	9.2 ± 3.73	<0.001
Magnesium (mg/day)	502.2 ± 94.90	398.9 ± 64.30	286.3 ± 90.71	<0.001
Iron (mg/day)	36.7 ± 6.35	28.4 ± 4.71	20.0 ± 5.59	<0.001
Zinc (mg/day)	15.3 ± 2.29	12.7 ± 1.99	9.2 ± 2.85	<0.001
Selenium (μg/day)	68.3 ± 21.32	50.5 ± 16.40	41.8 ± 18.64	<0.001
SFA (g/day)	7.3 ± 2.96	5.9 ± 3.18	4.4 ± 2.69	0.003
MUFA (g/day)	7.6 ± 3.56	5.98 ± 3.02	4.3 ± 2.55	0.003
PUFA (g/day)	6.0 ± 3.09	3.9 ± 1.78	2.61 ± 1.51	<0.001
**Food groups (g/day)**				
Onions	5.6 ± 6.04	6.2 ± 15.56	1.9 ± 3.19	0.221
Ginger	0.5 ± 1.21	0.8 ± 1.65	0.7 ± 1.00	0.679
Garlic	0.9 ± 4.02	0.05 ± 0.15	0.03 ± 0.11	0.786
Green tea	51.5 ± 120.15	28.6 ± 112.10	0	0.051
Caffeine	10.0 ± 44.72	0	0	0.368

Data are presented as mean ± SD and medians (interquartile range, IQR). P-value was obtained from analysis of variance (ANOVA) for continuous variables, and Kruskal-Wallis tests were used for skewness distribution data. All nutrients have been adjusted for total energy intake using a residual method. SFA, Saturated fatty acid; MUFA, Monounsaturated fatty acid; PUFA, Polyunsaturated fatty acid.

### Baseline characteristics and inflammatory factors in different severity of AD

**Figure 1:**
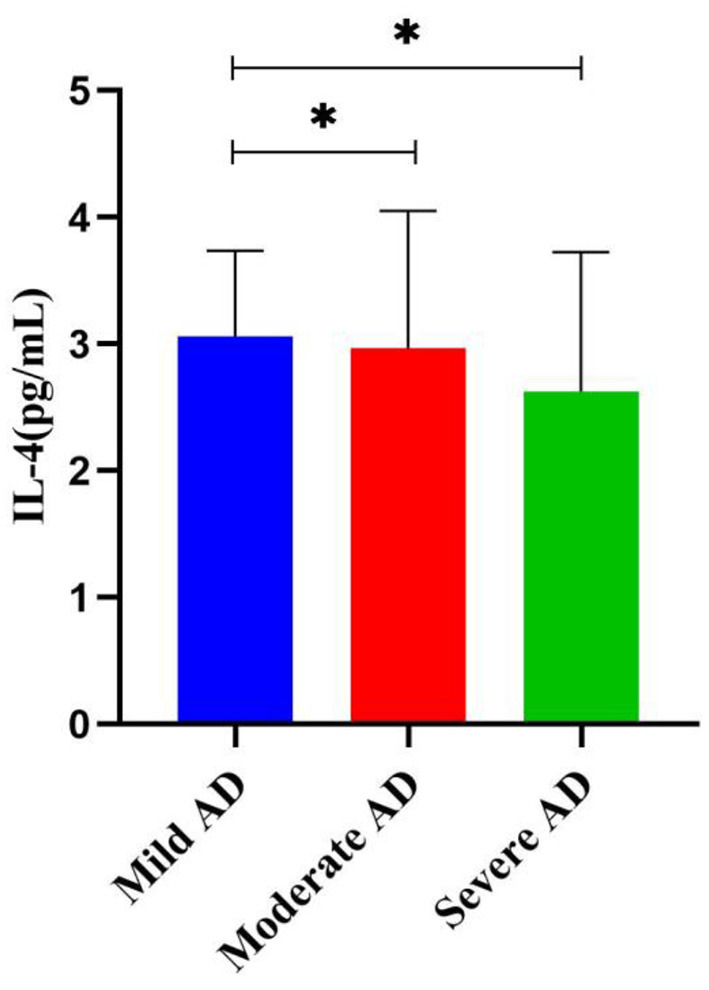
Differences in serum IL-4 levels in different AD groups (mean ± SEM). *p < 0.05.

Considering the progression of AD, we further analyzed the association between baseline characteristics and inflammatory factors with different severity of AD (Table 4), significant differences were observed only for the education level, MMSE scores, and IL-4 (*p* < 0.05). After pairwise comparison (Figure 1), the IL-4 levels in patients with moderate and severe AD were significantly higher than those in mild AD (*p* < 0.05).

**Table 4:** Baseline characteristics and inflammatory factors in different severity of AD.

**Variable**	**Mild AD (*n* = 26)**	**Moderate AD (*n* = 29)**	**Severe AD (*n* = 5)**	**F/χ2**	***p*-Value**
	**21–26**	**11–20**	**0–10**		
Age (years)	75.5 ± 1.76	75.6 ± 2.13	68.6 ± 2.37	1.085	0.345
BMI (kg/m^2^)	22.8 ± 0.75	23.5 ± 0.53	24.8 ± 3.96	0.892	0.416
Energy intake (kcal/day)	1,958.3 ± 132.68	1,962.83 ± 86.91	1,835.8 ± 188.63	0.113	0.894
DII score	0.8 ± 0.33	1.11 ± 0.21	1.36 ± 0.16	0.523	0.595
Sex					
Male	10 (38.5)	14 (48.3)	3 (60.0)	1.029	0.598
Female	16 (61.5)	15 (51.7)	2 (40.0)		
Education level, n (%)				36.472	<0.001
None	0 (0.0)	9 (31.0)	3 (60.0)		
Primary school	1 (3.8)	12 (41.4)	2 (40.0)		
Middle school	12 (46.2)	1 (3.4)	0 (0.0)		
High school or higher	13 (50.0)	7 (24.1)	0 (0.0)		
Income (yuan), n (%)				3.635	0.726
<3,000	4 (16.0)	8 (27.6)	0 (0.0)		
3,000–5,000	4 (16.0)	4 (13.8)	1 (20.0)		
5,000–10,000	6 (24.0)	9(31.0)	2 (40.0)		
>10,000	11 (44.0)	8 (27.6)	2 (40.0)		
Smoking status, n (%)				3.969	0.410
Never	19 (76.0)	22 (75.9)	2 (40.0)		
Former	4 (16.0)	3 (10.3)	2 (40.0)		
Current	2 (8.0)	4 (13.8)	1 (20.0)		
Alcohol status, *n* (%)				8.058	0.234
Current	1 (3.8)	2 (6.9)	1 (20.0)		
Former	8 (30.8)	6 (20.7)	1 (20.0)		
Never	17 (65.4)	21 (72.4)	3 (60.0)		
Diabetes, n (%)				4.079	0.130
Yes	7 (29.2)	3 (10.3)	2 (40.0)		
No	17 (70.8)	26 (89.7)	3 (60.0)		
Hypertension, n (%)				1.927	0.382
Yes	11 (45.8)	8 (27.6)	2 (40.0)		
No	13 (54.2)	21 (72.4)	3 (60.0)		
Hyperlipidemia, *n* (%)				3,155	0.207
Yes	5 (20.8)	2(6.9)	0 (0.0)		
No	19(79.2)	27 (93.1)	5 (100.0)		
Coronary heart disease, *n* (%)				0.448	0.800
Yes	2 (8.3)	2 (6.9)	0 (100.0)		
No	22 (91.7)	27 (93.1)	0 (0.0)		
Cerebrovascular disease, *n* (%)				0.309	0.857
Yes	3 (12.5)	5 (17.2)	1 (20.0)		
No	21 (87.5)	24 (82.8)	4 (80.0)		
Diastolic blood pressure (mmHg)	77.04 ± 2.30	80.08 ± 1.81	85.0 ± 4.65	1.429	0.249
Physical activity (METs × h/wk), medians (IQR)	37.1 (11.55–71.48)	26.95 (14.55–83.30)	58.1 (10.1–180.42)	0.146	0.930
Systolic blood pressure (mmHg)	131.04 ± 4.56	129.92 ± 3.65	125.4 ± 5.63	0.165	0.848
MMSE	22.46 ± 0.23	17.62 ± 0.33	8.40 ± 1.95	191.050	<0.001
IL-1β (pg/mL)	3.59 ± 0.16	3.53 ± 0.11	3.47 ± 0.41	0.094	0.911
IL-4 (pg/mL)	2.91 ± 0.11	3.32 ± 0.11	3.62 ± 0.16	5.118	0.009
IL-10 (pg/mL)	5.61 ± 0.19	6.00 ± 0.15	6.02 ± 0.30	1.395	0.256
IL-12 (pg/mL)	3.06 ± 0.13	2.96 ± 0.20	2.62 ± 0.50	0.461	0.633
TNF-α (pg/mL)	4.09 ± 0.99	3.88 ± 0.99	4.11 ± 0.26	1.196	0.310
hs-CRP (mg/L)	1.93 ± 1.28	2.01 ± 0.10	2.25 ± 0.15	0.678	0.512
IL-6 (pg/mL)	2.73 ± 0.13	2.88 ± 0.10	2.74 ± 0.28	0.448	0.641
C3 (μg/mL)	5.60 ± 0.16	5.88 ± 0.11	5.70 ± 0.45	0.961	0.388

Data are presented as mean ± SD and obtained from analysis of variance (ANOVA).

### The α- and β-diversity of the oral and gut microbiomes in participants with AD

**Figure 2:**
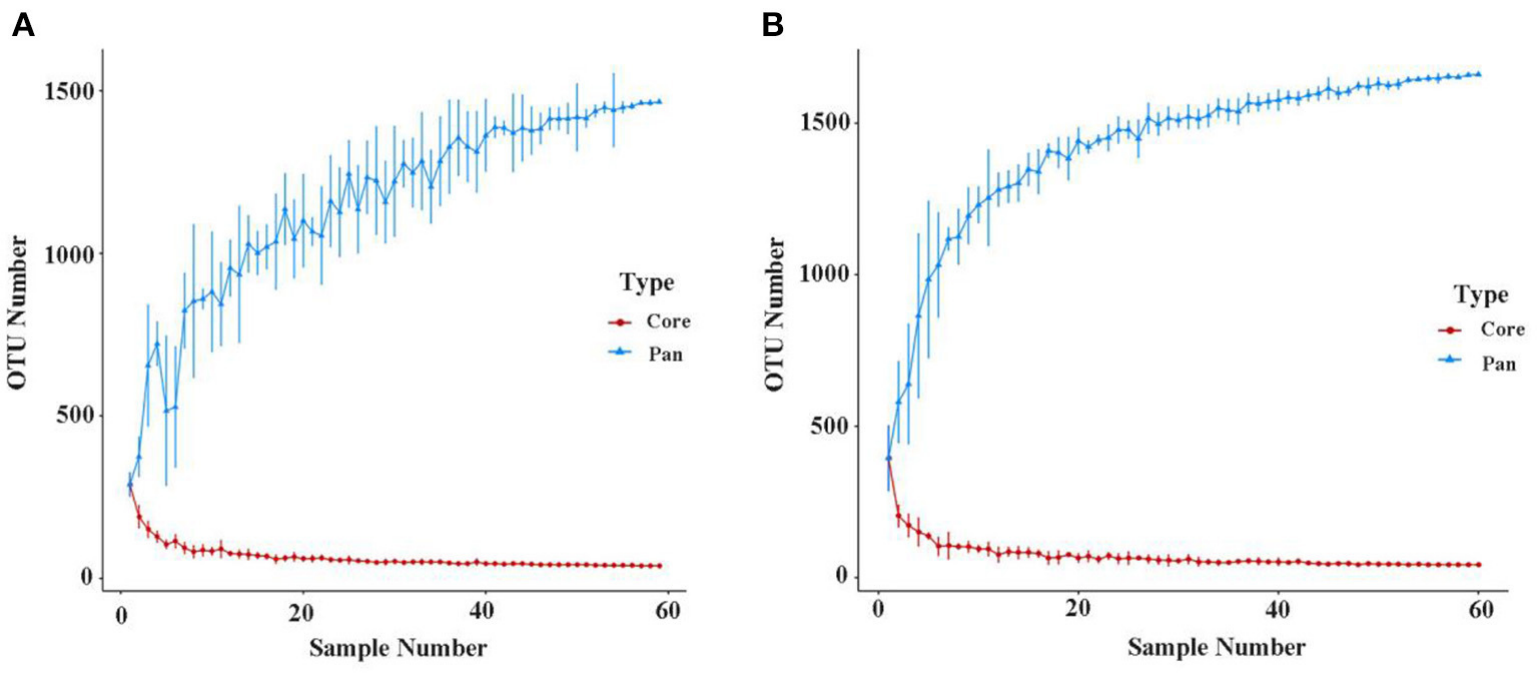
Pan/Core OTU species analysis of the oral **(A)** and gut microbiomes **(B)**. The Pan/Core plot reflects the rate of emergence of species under continuous sampling. The curve flattens out as the sample size increases, indicating that species don't increase significantly with sample size.

**Figure 3:**
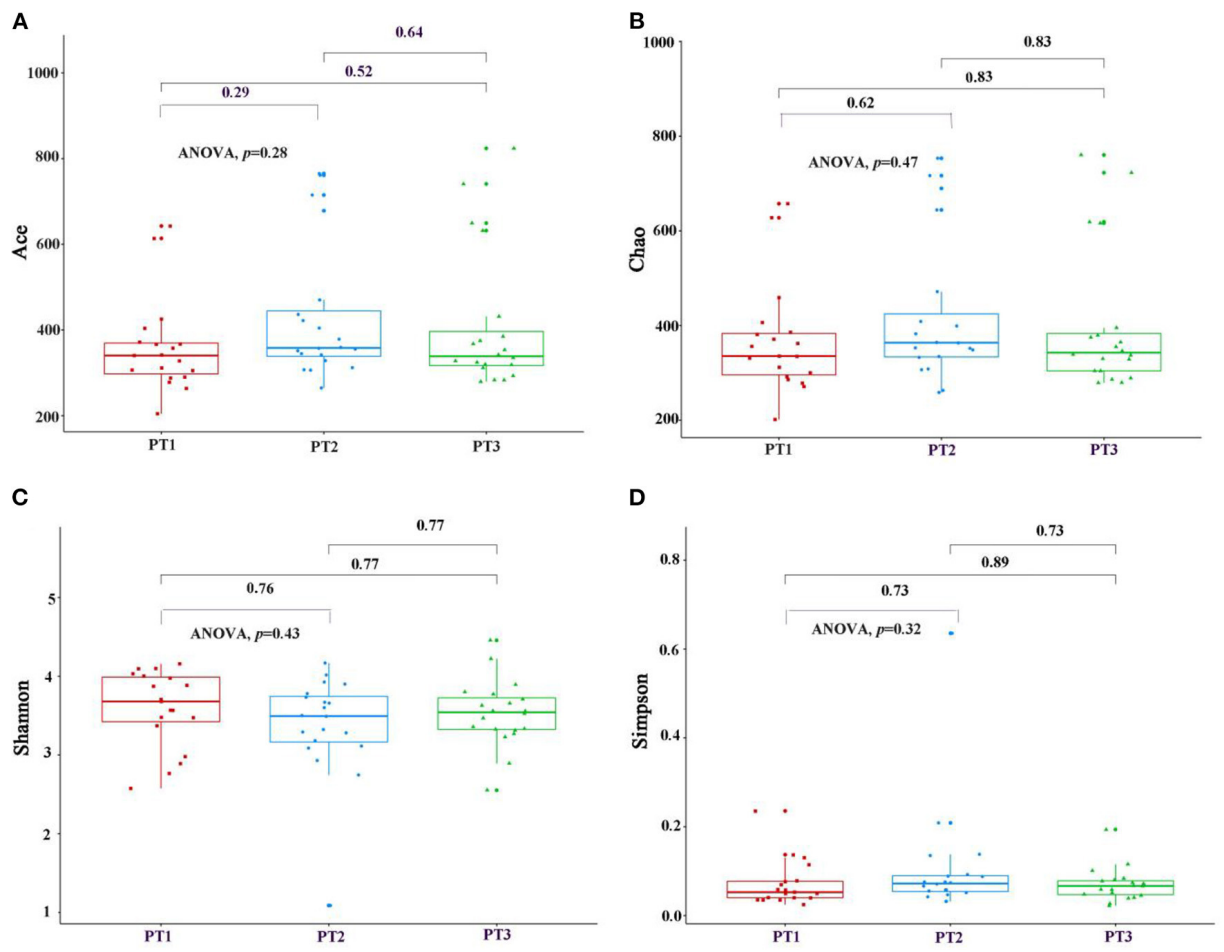
The oral microbial α-diversity, as assessed with ACE **(A)**, Chao **(B)**, Shannon **(C)**, and Simpson **(D)** indexes among three groups. PT1 (tertile 1) means the oral microbiomes of the most anti-inflammatory diet group, PT2 (tertile 2) means the oral microbiomes of the no anti-inflammatory/pro-inflammatory diet group, and PT3 (tertile 3) means the oral microbiomes of the most pro-inflammatory diet group.

**Figure 4:**
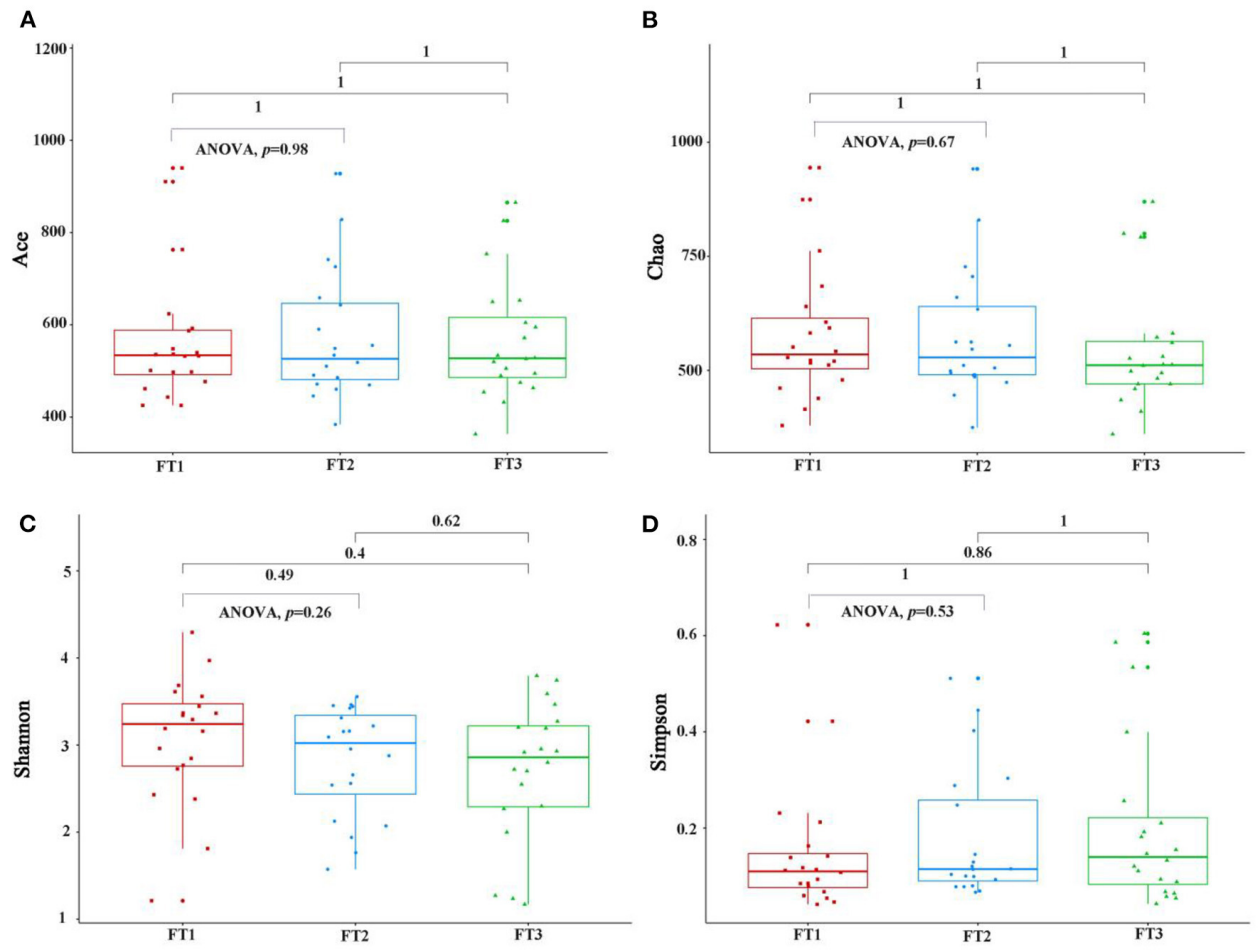
The gut microbial α-diversity, as assessed with the ACE **(A)**, Chao **(B)**, Shannon **(C)**, and Simpson **(D)** indexes among three groups. FT1 (tertile 1) means the gut microbiomes of the most anti-inflammatory diet group, FT2 (tertile 2) means the gut microbiomes of the no anti-inflammatory/pro-inflammatory diet group, and FT3 (tertile 3) means the gut microbiomes of the most pro-inflammatory diet group).

**Figure 5:**
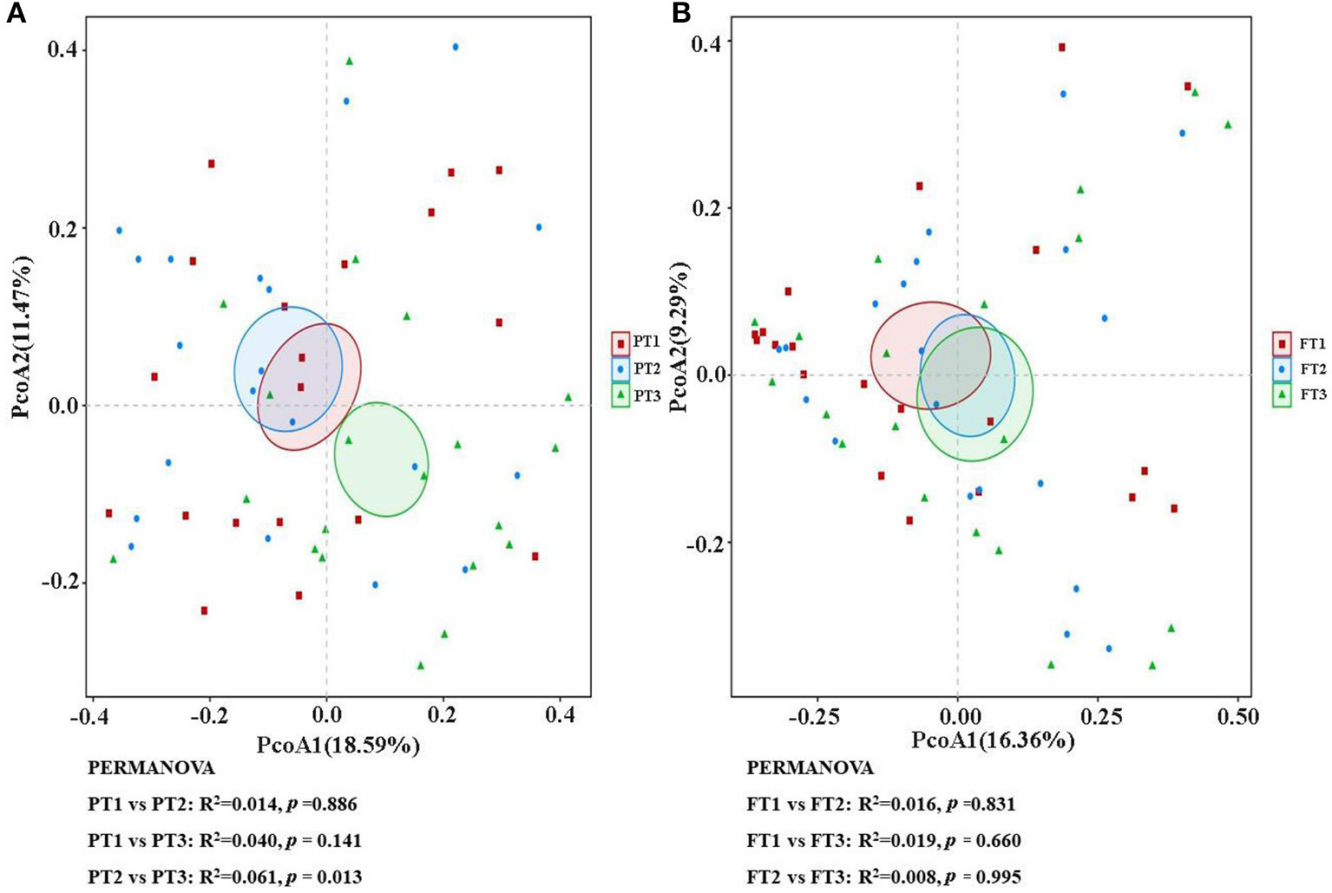
The β-diversity analysis of the oral **(A)** and gut **(B)** microbiomes among different DII tertiles in patients with AD. The PCoA based on the Bray–Curtis of β-diversity analysis is presented. PCoA, principal coordinate analysis.

As shown in Figure 2, when the number of samples reached 60, the number of species observed was nearly parallel, indicating that the sample size of our experiment was sufficient. There were no significant differences among the three groups in the oral microbiomes in the Shannon, Simpson, Chao, or ACE indices (Figure 3, *p* > 0.05). Furthermore, there were no differences in the α-diversity of gut microbiomes across the different DII tertiles in patients with AD (Figure 4, *p* > 0.05). Interestingly, the PCoA based on weighted UniFrac distances showed (Figure 5) that the oral microbiomes of PT1 largely overlapped with the microbial distribution of PT2, whereas there were statistical differences in the microbial distribution of PT2 and PT3 (PERMANOVA, Bray–Curtis: PT2 vs PT3, R^2^ = 0.061, *p* = 0.013). As for the gut microbiomes, no differences were found among the FT1, FT2, and FT3 groups.

### Alterations of the oral and gut microbiomes in participants with AD

**Figure 6:**
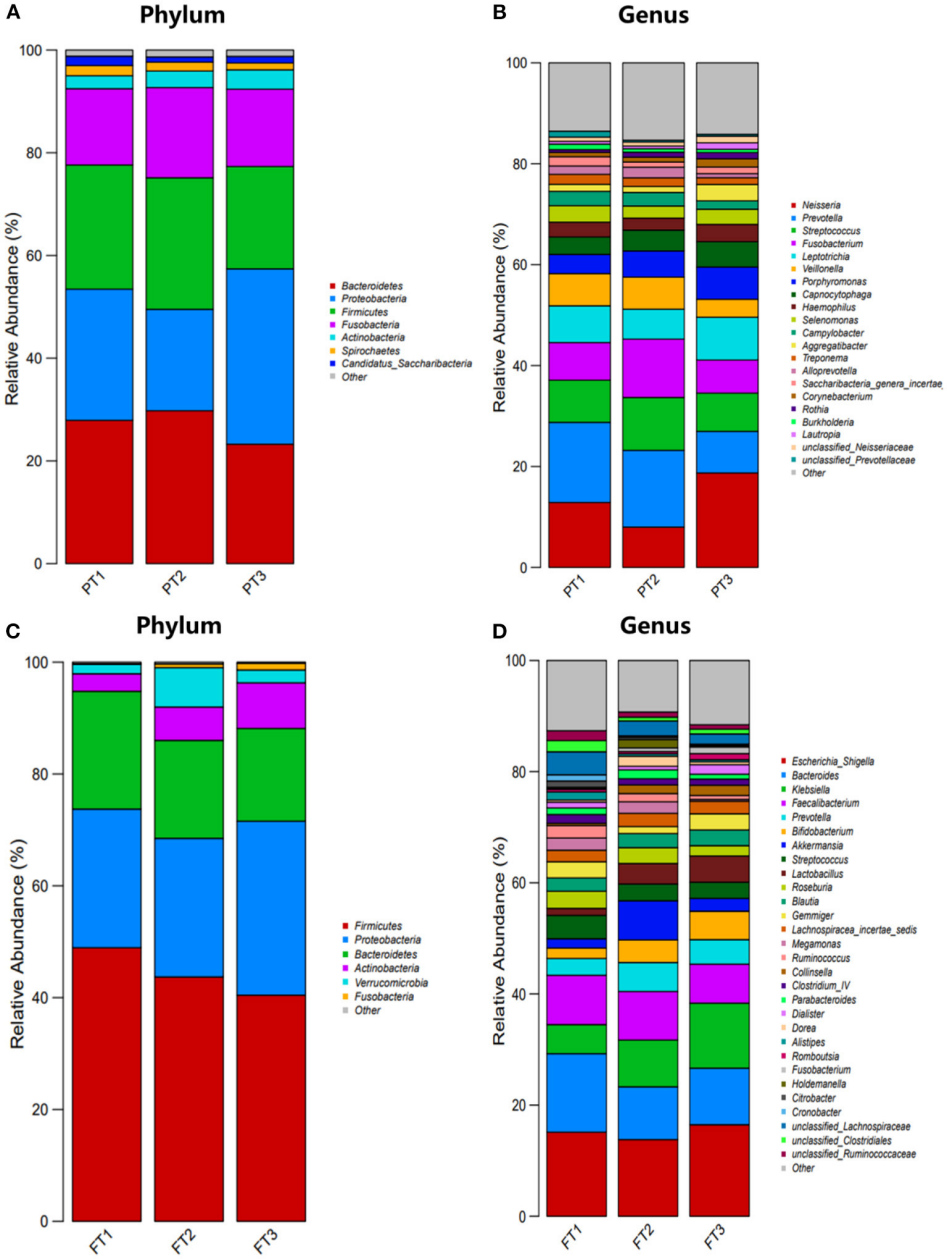
Distribution of oral and gut microbiomes between the most anti-inflammatory diet group, no anti-inflammatory/pro-inflammatory diet group, and the most pro-inflammatory diet group. **(A,B)** represent the relative abundance of oral microbiomes at the phylum level and genus level, respectively; **(C,D)** represent the relative abundance of gut microbiomes at the phylum level and genus level, respectively.

The relative abundance at the phylum and genus levels revealed similar overall microbiome composition among the three groups in the oral microbiomes. The most abundant phyla were *Bacteroidetes, Proteobacteria, Firmicutes, Fusobacteria*, and *Actinobacteria*; these five phyla accounted for more than 95% of the total abundance of all the species (Figure 6A). At the genus level, *Neisseria, Prevotella, Streptococcus, Fusobacterium*, and *Leptotrichia* were the top five most abundant bacterial taxa, as shown in Figure 6B. We also found that in the gut microbiomes, *Firmicutes, Proteobacteria, Bacteroidetes, Actinobacteria*, and *Verrucomicrobia* were identified as the most abundant sequences at the phylum level (Figure 6C). At the genus level, *Escherichia, Shigella, Bacteroides, Klebsiella, Faecalibacterium*, and *Prevotella* were the most abundant bacterial taxa, as shown in Figure 6D.

**Figure 7:**
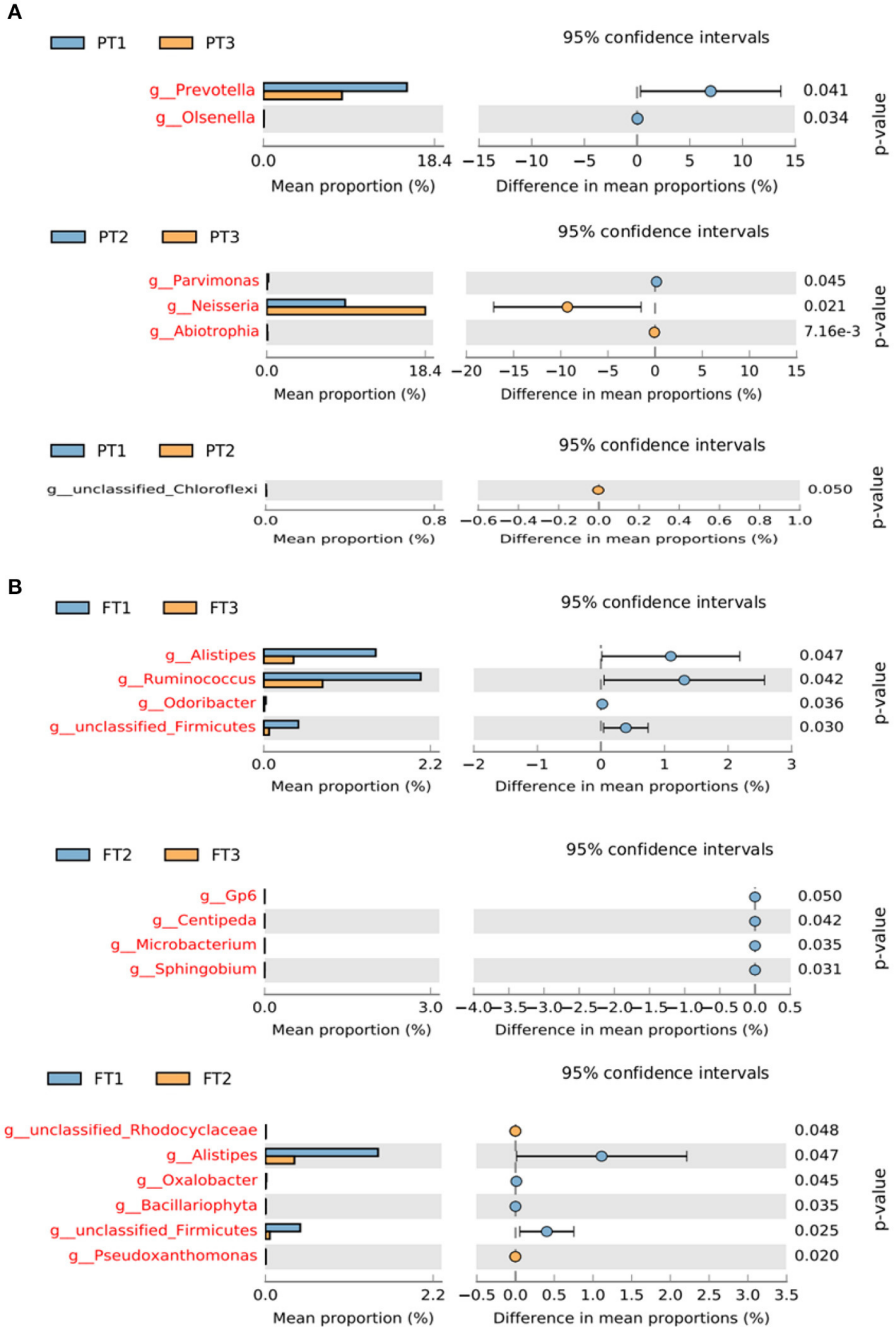
Differences in the relative abundance of bacterial genera in the oral **(A)** and gut **(B)** microbiomes from the most anti-inflammatory diet group and the most pro-inflammatory diet group. The left of the figure shows the differential species between the two groups, and the right shows the difference in the mean proportions of the target species (*p* < 0.05).

The STAMP results showed that there were no significant differences in the percent relative abundance of any phylum among the groups. At the genus level, the abundance of *Prevotella* and *Olsenella* in the oral microbiomes was much higher in PT1 (the most anti-inflammatory diet group) than in PT3 (the most pro-inflammatory diet group). The differences in the relative abundance of *Abiotrophia, Neisseria*, and *Parvimonas* between the PT2 (no anti-inflammatory/pro-inflammatory diet group) and PT3 groups were statistically significant (Figure 7A). In addition, the abundance of genera *Alistipes, Ruminococcus, Odoribacter*, and unclassified *Firmicutes* in the gut microbiomes were lower in the FT3 group than in the FT1 group. The differences in the relative abundance of *Sphingobium, Microbacterium, Centipeda*, and *Gp6* were statistically significant between the FT2 and FT3 groups, and the other branching genera *Pseudoxanthomonas, Firmicutes, Bacillariophyta, Oxalobacter, Alistipes*, and *Rhodocyclaceae* were also found to be significantly different between the FT1 and FT2 groups (Figure 7B).

To further explore the differences in oral and gut microbiomes between the anti-inflammatory diet and pro-inflammatory diet groups, we used LEfSe (linear discriminant analysis score cut off >2.0) analysis to identify the key taxa responsible for the differences in the compositions of the oral and gut microbiota. However, there were no significant differences among the three groups of patients with AD (Supplementary material for details).

### Association between inflammatory markers and microbiota in patients with AD

**Figure 8:**
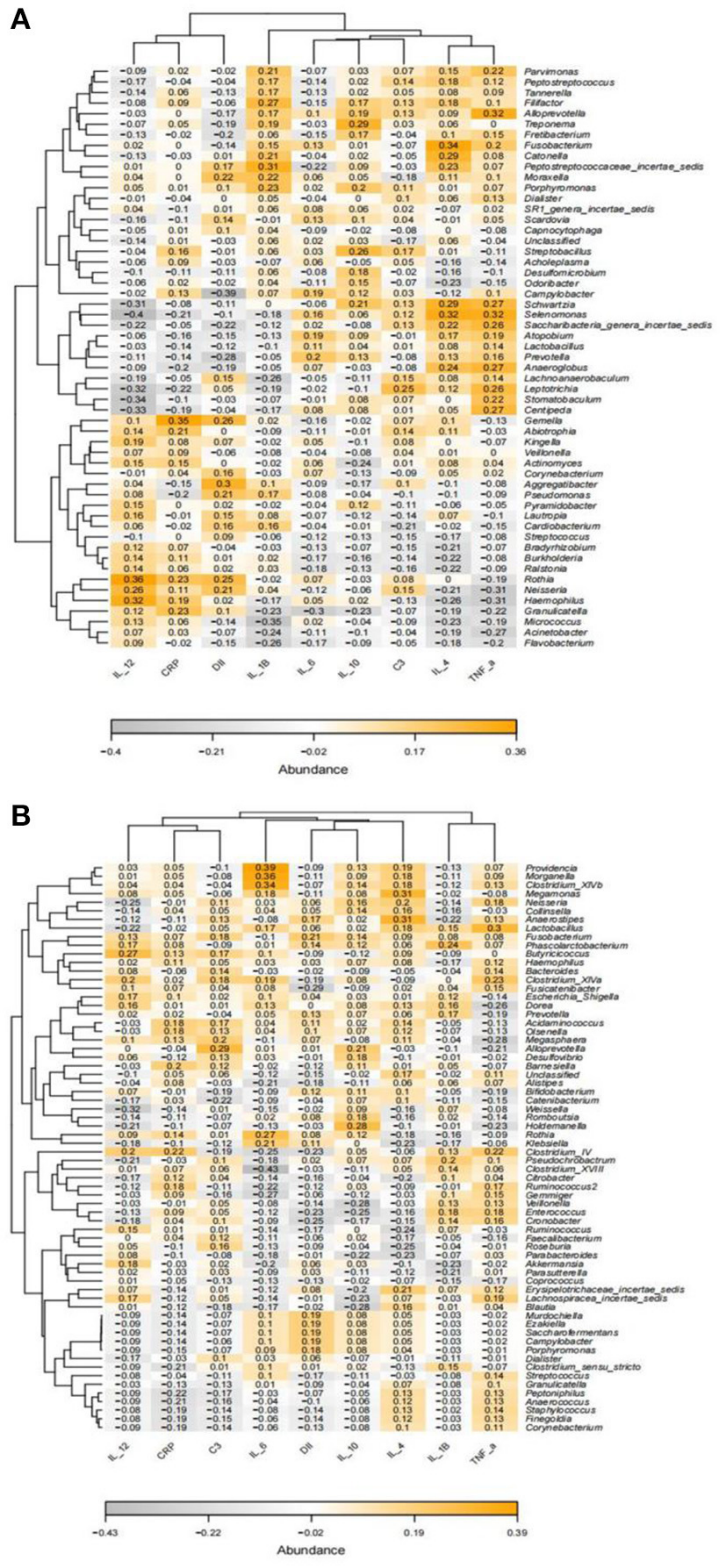
Heatmap of the correlation between Spearman's analysis of inflammatory markers and the relative abundance of oral **(A)** and gut microbiota **(B)** at the genus level. Orange and gray represent positive and negative correlations, respectively, with color intensity proportional to the degree of association between indices, and numbers represent r values.

Spearman correlation analysis showed significant correlations between inflammatory markers and specific oral and gut microbiota (Figure 8). In the analysis of oral microorganisms, IL-4 was positively correlated with *Fusobacterium* (*p* = 0.008, r = 0.34) and *Selenomonas* (*p* = 0.012, r = 0.32); IL-1B was positively correlated with *Peptostreptococcaceae_incertae_sedis* (*p* = 0.018, r = 0.31); IL-12 was positively correlated with *Haemophilus* (*p* = 0.013, r = 0.32) and *Rothia* (*p* = 0.005, r = 0.36) and negatively correlated with *Selenomonas* (*p* = 0.001, r = −0.4); TNF-α was positively correlated with *Alloprevotella* (*p* = 0.013, r = 0.32) and *Selenomonas* (*p* = 0.014, 0.32) and negatively correlated with *Haemophilus* (*p* = 0.018, r = −0.31); CRP was positively correlated with *Gemella* (*p* = 0.006, r = 0.35); and IL-6 was negatively correlated with *Granulicatella* (*p* = 0.022, r = −0.3). Furthermore, in the analysis of intestinal microorganisms, IL-4 was positively correlated with *Megamonas* (*p* = 0.016, r = 0.31) and *Anaerostipes* (*p* = 0.014, r = 0.31); IL-12 was negatively correlated with *Weissella* (*p* = 0.013, r = −032); TNF-α was positively correlated with *Lactobacillus* (*p* = 0.019, r = 0.3); and IL-6 was positively correlated with *Clostridium_XlVb* (*p* = 0.008, r = 0.34), *Morganella* (*p* = 0.005, r = 0.36), and Providencia (*p* = 0.002, r = 0.39).

## Discussion

In the present study, no association was found between DII and inflammatory markers, but IL-4 was related with the severity of AD. More importantly, we found no significant differences in diversity among the groups stratified by DII, except for differences in the β-diversity of the oral cavity. At the same time, the abundance of microbial composition in the oral cavity and intestine was different between the anti-inflammatory and pro-inflammatory diet groups. In addition, oral and gut microbiota is considered associated with markers of inflammation.

Although DII has been demonstrated to be associated with inflammatory markers, no association was found in the present study. Similar findings were found in several studies that did not find an association with hs-CRP (54, 55). This may be related to memory bias and homogeneity as well as to the small number of components included. Firstly, the FFQ relies on the memory of the respondents and there may be recall bias. Secondly, the inflammatory index profile of the AD population is possible similar. Finally, only 25 food components were counted in this study; however, the richness and diversity of foods in China may underestimate inflammatory levels. And our study found a higher intake of anti-inflammatory antioxidant foods in the anti-inflammatory diet group. A prospective study found that a diet characterized by high consumption of anti-inflammatory foods (monounsaturated fatty acids, polyunsaturated fatty acids, antioxidant foods) was associated with lower DII scores (56). The anti-inflammatory dietary composition in this study is similar to that of the Mediterranean diet. It is well known that the Mediterranean diet consists mainly of high-quality fatty acids, low in saturated fatty acids and cholesterol, an appropriate ratio of polyunsaturated fatty acids omega-3 to omega-6, and high in carotenoids and fiber, which have anti-inflammatory effects and can significantly reduce inflammation levels (57, 58). In addition, we found that only IL-4 of the eight inflammatory markers (IL-1β, IL-10, IL-6, IL-12, TNF-α, hs-CRP, and C3) was associated with the severity of AD, and IL-4 levels were higher in patients with severe AD. It has been proposed that inflammatory markers appear early in AD and that inflammatory levels decline with disease progression (59). The study shows that significantly higher levels of IL-10, IL-1β, IL-4, and IL-2 in mild cognitive impairment (MCI) groups, while there was no significant difference in inflammatory markers between dementia, suggesting that peripheral inflammation may occur in the early stages of AD (60). IL-4 has been reported to have anti-inflammatory effects and reduce inflammation production (61, 62). In conjunction with this study, it is boldly speculated that IL-4 may be associated with lower levels of inflammation in AD. Although no other inflammatory markers were found to be associated with AD severity in this study, this may be due to the lack of a healthy control group and the small sample size. Further validation with larger sample size and well-designed studies is needed in the future.

There are no significant differences in diversity among the groups stratified by DII, except for differences in the β-diversity of the oral cavity, suggesting that the community structure of the oral cavity may be influenced by inflammatory diet. The abundance of microbial composition in the oral cavity and intestine was altered. Anderson et al. (63) also observed differences in β-diversity in the oral microbiome across levels of intake of total carbohydrates, fiber, sucrose, and galactose (64). Another study showed that β-diversity was significantly altered in patients with gingivitis on a nitrate-rich diet compared to that in healthy participants (65). These findings show that differences in the distribution of abundance of oral microbes are affected significantly by foods. Little is currently known about the significance of this change, and more mechanistic studies are thus needed to explore its role. A study by Zheng et al. showed results similar to ours, in which DII was not associated with gut microbial diversity but was associated with gut microbial composition (66). Tian et al. also found that diets with high DII scores were associated with the presence of specific microbes but not the overall diversity of the gut microbiome (67). Other studies comparing the relationship between different diets and gut microbial diversity have had similar findings, with diet affecting specific microflora composition and having a lower or no effect on overall diversity (68, 69). Furthermore, oral-gut α-diversity was not associated with DII and may be related to unmeasured confounding factors, individual variability, and small sample size. Reduced microbial diversity has been reported in patients with AD, and microorganisms are stable over a time frame (6), which may diminish the role of dietary inflammation.

This study revealed a decreased abundance of *Prevotella* and *Olsenella* in the oral cavity of patients with patients consuming the most pro-inflammatory diet and reduced intestinal abundance of *Alistipes, Ruminococcus, Odoribacter*, and unclassified *Firmicutes* compared to those in the most anti-inflammatory diet group. *Prevotella* and *Olsenella* are normal in the oral cavity (70, 71) and produce SCFA metabolites like butyrate (72). *Prevotella* has been reported to be associated with a fiber-rich diet (73), which, in turn, is associated with better cognition (74). In addition, studies have reported that *Odoribacter* was reduced in the gut of AD compared to the normal population (75) and the abundance of *Alistipes* was reduced in the gut of mild cognitive impairment population (76). It is worth mentioning that the most anti-inflammatory diet led to a significant increase in the abundance of *Ruminococcus* and *Prevotella*, the main genera that produce SCFAs. SCFAs have important regulatory effects on the blood-brain barrier and the nervous system and are associated with the development of AD (77–79). Furthermore, *Ruminococcus* and *Prevotella* are closely associated with the intake of plant-based diets (17, 69, 80). Plant-based diets are well-known as being beneficial to overall health, including the prevention of AD (81, 82). In summary, it is shown that an anti-inflammatory diet may be associated with increased abundance of beneficial microbes in AD patients, especially microorganisms that produce anti-inflammatory compounds.

The study observed higher levels of pro-inflammatory cytokines (e.g., IL-6 and IL-1β) and reduced levels of the anti-inflammatory cytokine IL-10 in patients with cognitive dysfunction (83). Abundant *E. coli/Shigella* were positively correlated with IL-1β levels, whereas rectal eubacteria were negatively correlated with IL-1β levels and positively correlated with IL-10 levels. These findings suggest that intestinal components may drive peripheral inflammation, leading to brain amyloidosis and possibly neurodegeneration and cognitive symptoms in AD. Another study demonstrated that inflammation levels and intestinal flora may contribute to AD-related neuroinflammation (84). It is suggested that the altered inflammatory state in patients with AD may induce neuropathy by affecting the growth and reproduction of microorganisms within the host or indirectly by affecting the interactions between genera, causing microecological dysregulation. It is boldly speculated that the altered microbiota may be associated with a systemic inflammatory response.

To further support our speculation, we correlated systemic inflammatory markers with microbiota and found that a specific oral and gut microbial composition was associated with inflammatory markers. *Fusobacterium* and *Selenomonas* have been reported to be associated with unhealthy microbial characteristics in patients with gingival and periodontal disease, whereas *Fusobacterium* may be a major indicator of microbial transformation into inflammatory pathogenic bacteria (85, 86) and a potential biomarker of periodontitis and gingivitis episodes (87, 88). In addition, *Rothia, Haemophilus*, and *Alloprevotella* are normal in the oral cavity (70); *Anaerostipes* and *Weissella* are common in the intestine, with *Anaerostipes* facilitating acetate formation (89, 90), whereas *Weissella* is an emerging genus with potential health benefits, capable of inhibiting pathogenic microbial growth through the production of bacteriocins (91, 92). One study found *Morganella* enrichment and reduced abundance of *Megamonas* in patients with AD (93). *Megamonas* has been shown to produce SCFAs that favor host metabolism and slow the development of AD (94), whereas *Clostridium_XlV* has also been found to be associated with cognitive impairment (95). *Providencia* is likely to promote inflammation (96, 97). *Lactobacillus* is the most abundant in the human intestine, and experimental studies in animals and humans have shown that the administration of *Lactobacillus* probiotics facilitates the recovery of dysregulated intestinal flora and improves cognitive function (98–100). The data above highlight the inflammatory markers that are associated with normal microorganisms in the oral cavity and intestine. It has been proposed that an enhanced inflammatory response allows for the conversion of non-pathogenic microorganisms to pathogenic microorganisms (101). Corrêa et al. (102) found a positive correlation between the presence of pathogenic bacteria and the level of systemic inflammation as indicated by CRP levels. A study by Xiao et al. (103) demonstrated that the pathogenicity of oral microflora was diminished when IL-17 was inhibited. Combined with the analysis of the results of this study, increased inflammation may be associated with the conversion of the normal microbiota of the host oral-intestinal tract to pathogenic microorganisms.

Our research is innovative and comprehensive, analyzing for the first time the relationship between DII and markers of systemic inflammation and exploring its relationship with systemic inflammation by analyzing the microbiological characteristics of the oral cavity and gut in patients with AD. Nevertheless, there are shortcomings. Firstly, this was a cross-sectional study, and no causal conclusions could be drawn. Secondly, the small sample size included only the AD population, making the findings not generalizable. And it would be interesting to explore the relationship between DII scores, energy intake and AD severity, this can be explored in the future in a larger sample size. In addition, we did not include a control group in this study. As a result, it is unclear whether our findings can apply to all older adults. Finally, the microbial community in this study was down only to the genus level, and a more in-depth species analysis was not possible. No metabolic function analysis was performed.

## Conclusions

Anti-inflammatory diets seem to be associated with increased abundance of beneficial microbes in the oral-gut axis in patients with AD, whereas microbes are related with specific inflammatory markers and inflammation accumulation may drive a shift from normal microbial composition to pathogenicity. Therefore, it is proposed that targeting the modulation of oral and gut microbiota, especially the improvement of SCFA-producing bacteria and reduction of pathogens, may be an effective and important strategy for the treatment of inflammatory disorders. More long-term, large-sample follow-up studies based on AD populations are needed in the future to explore the mechanistic role of dietary inflammation plays with oral-gut microbiota and systemic inflammation to provide a basis for precise dietary intervention strategies.

## Data availability statement

The datasets presented in this study can be found in online repositories. The names of the repository/repositories and accession number(s) can be found below: NCBI; PRJNA851957.

## Ethics statement

The studies involving human participants were reviewed and approved by Ethics Committee of Fujian Provincial Hospital (Ref No. K2020-09-025). The patients/participants provided their written informed consent to participate in this study.

## Author contributions

LC, BW, and JL contributed to conception and design of the study. LC wrote the first draft of the manuscript. BW and JL performed the statistical analysis. XW, XX, HC, XJ, PZ, XL, and ZH acquired the data. HL approved the final version of the manuscript. All authors contributed to manuscript revision, read, and approved the submitted version.

## Funding

This research was funded by the Natural Science Foundation of Fujian Province, China (Grant No. 2019J01501) and Fujian Provincial Health Technology Project of middle-aged and young backbone Talents Training Project, China (Grant No. 2020GGA011). The funding sources had no role in the design and execution of the study, the collection, management, analysis, interpretation of the data, the preparation, review, approval of the manuscript, and the decision to submit the manuscript for publication.

## Conflict of interest

The authors declare that the research was conducted in the absence of any commercial or financial relationships that could be construed as a potential conflict of interest.

## Publisher's note

All claims expressed in this article are solely those of the authors and do not necessarily represent those of their affiliated organizations, or those of the publisher, the editors and the reviewers. Any product that may be evaluated in this article, or claim that may be made by its manufacturer, is not guaranteed or endorsed by the publisher.

## References

[1] Alzheimer's disease facts and figures. Alzheimers Dement. (2021) 17:327–406. 10.1002/alz.12328 33756057

[2] Prince M Bryce R Albanese E Wimo A Ribeiro W Ferri CP. The global prevalence of dementia: a systematic review and metaanalysis. Alzheimer Dement. (2013) 9:63-75.e2. 10.1016/j.jalz.2012.11.007 23305823

[3] Jia L Du Y Chu L Zhang Z Li F Lyu D. Prevalence, risk factors, and management of dementia and mild cognitive impairment in adults aged 60 years or older in China: a cross-sectional study. Lancet Public Health. (2020) 5:e661–e71. 10.1016/S2468-2667(20)30185-7 33271079

[4] Bhatti GK Reddy AP Reddy PH Bhatti JS. Lifestyle modifications and nutritional interventions in aging-associated cognitive decline and Alzheimer's Disease. Front Aging Neurosci. (2019) 11:369. 10.3389/fnagi.2019.00369 31998117 PMC6966236

[5] Hill E Goodwill AM Gorelik A Szoeke C. Diet and biomarkers of Alzheimer's disease: a systematic review and meta-analysis. Neurobiol Aging. (2019) 76:45–52. 10.1016/j.neurobiolaging.2018.12.008 30682676

[6] Claesson MJ Cusack S O'Sullivan O Greene-Diniz R de Weerd H Flannery E. Composition, variability, and temporal stability of the intestinal microbiota of the elderly. Proc Natl Acad Sci U S A. (2011) 108 (Suppl 1):4586–91. 10.1073/pnas.1000097107 20571116 PMC3063589

[7] Biagi E Nylund L Candela M Ostan R Bucci L Pini E. Through ageing, and beyond: gut microbiota and inflammatory status in seniors and centenarians. PLoS ONE. (2010) 5:e10667. 10.1371/journal.pone.0010667 20498852 PMC2871786

[8] Roberts RO Geda YE Knopman DS Boeve BF Christianson TJ Pankratz VS. Association of C-reactive protein with mild cognitive impairment. Alzheimer Dement. (2009) 5:398–405. 10.1016/j.jalz.2009.01.025 19751919 PMC2851170

[9] Magaki S Mueller C Dickson C Kirsch W. Increased production of inflammatory cytokines in mild cognitive impairment. Exp Gerontol. (2007) 42:233–40. 10.1016/j.exger.2006.09.015 17085001 PMC1868444

[10] Noble JM Manly JJ Schupf N Tang MX Mayeux R Luchsinger JA. Association of C-reactive protein with cognitive impairment. Arch Neurol. (2010) 67:87–92. 10.1001/archneurol.2009.308 20065134 PMC4426905

[11] Bronzuoli MR Iacomino A Steardo L Scuderi C. Targeting neuroinflammation in Alzheimer's disease. J Inflamm Res. (2016) 9:199–208. 10.2147/JIR.S86958 27843334 PMC5098782

[12] Zhang M Zhao D Zhou G Li C. Dietary pattern, gut microbiota, and Alzheimer's Disease. J Agric Food Chem. (2020) 68:12800–9. 10.1021/acs.jafc.9b08309 32090565

[13] Vogt NM Kerby RL Dill-McFarland KA Harding SJ Merluzzi AP Johnson SC. Gut microbiome alterations in Alzheimer's disease. Sci Rep. (2017) 7:13537. 10.1038/s41598-017-13601-y 29051531 PMC5648830

[14] Ling Z Zhu M Yan X Cheng Y Shao L Liu X. Structural and functional dysbiosis of fecal microbiota in chinese patients with Alzheimer's Disease. Front Cell Dev Biol. (2020) 8:634069. 10.3389/fcell.2020.634069 33614635 PMC7889981

[15] Giau VV Wu SY Jamerlan A An SSA Kim SY Hulme J. Gut Microbiota and Their Neuroinflammatory Implications in Alzheimer's Disease. Nutrients. (2018) 10:1765. 10.3390/nu10111765 30441866 PMC6266223

[16] Goyal D Ali SA Singh RK. Emerging role of gut microbiota in modulation of neuroinflammation and neurodegeneration with emphasis on Alzheimer's disease. Prog Neuropsychopharmacol Biol Psychiatry. (2021) 106:110112. 10.1016/j.pnpbp.2020.110112 32949638

[17] Tomova A Bukovsky I Rembert E Yonas W Alwarith J Barnard ND. The effects of vegetarian and vegan diets on gut microbiota. Front Nutr. (2019) 6:47. 10.3389/fnut.2019.00047 31058160 PMC6478664

[18] Pistollato F Sumalla Cano S Elio I Masias Vergara M Giampieri F Battino M. Role of gut microbiota and nutrients in amyloid formation and pathogenesis of Alzheimer disease. Nutr Rev. (2016) 74:624–34. 10.1093/nutrit/nuw023 27634977

[19] Wieckowska-Gacek A Mietelska-Porowska A Wydrych M Wojda U. Western diet as a trigger of Alzheimer's disease: From metabolic syndrome and systemic inflammation to neuroinflammation and neurodegeneration. Ageing Res Rev. (2021) 70:101397. 10.1016/j.arr.2021.101397 34214643

[20] Wieckowska-Gacek A Mietelska-Porowska A Chutorański D Wydrych M Długosz J Wojda U. Western diet induces impairment of liver-brain axis accelerating neuroinflammation and amyloid pathology in Alzheimer's Disease. Front Aging Neurosci. (2021) 13:654509. 10.3389/fnagi.2021.654509 33867971 PMC8046915

[21] Agus A Denizot J Thévenot J Martinez-Medina M Massier S Sauvanet P. Western diet induces a shift in microbiota composition enhancing susceptibility to Adherent-Invasive E. coli infection and intestinal inflammation. Sci Rep. (2016) 6:19032. 10.1038/srep19032 26742586 PMC4705701

[22] Hildebrandt MA Hoffmann C Sherrill-Mix SA Keilbaugh SA Hamady M Chen YY. High-fat diet determines the composition of the murine gut microbiome independently of obesity. Gastroenterology. (2009) 137:1716–24.e1-2. 10.1053/j.gastro.2009.08.042 19706296 PMC2770164

[23] Marizzoni M Cattaneo A Mirabelli P Festari C Lopizzo N Nicolosi V. Short-chain fatty acids and lipopolysaccharide as mediators between gut dysbiosis and amyloid pathology in Alzheimer's Disease. J Alzheimers Dis. (2020) 78:683–97. 10.3233/JAD-200306 33074224

[24] Jena PK Sheng L Di Lucente J Jin LW Maezawa I Wan YY. Dysregulated bile acid synthesis and dysbiosis are implicated in Western diet-induced systemic inflammation, microglial activation, and reduced neuroplasticity. FASEB J. (2018) 32:2866–77. 10.1096/fj.201700984RR 29401580 PMC5901391

[25] Bifulco M. Mediterranean diet: the missing link between gut microbiota and inflammatory diseases. Eur J Clin Nutr. (2015) 69:1078. 10.1038/ejcn.2015.81 26014263

[26] Gu Y Luchsinger JA Stern Y Scarmeas N. Mediterranean diet, inflammatory and metabolic biomarkers, and risk of Alzheimer's disease. J Alzheimers Dis. (2010) 22:483–92. 10.3233/JAD-2010-100897 20847399 PMC3022949

[27] Lewis JD Chen EZ Baldassano RN Otley AR Griffiths AM Lee D. Inflammation, antibiotics, and diet as environmental stressors of the gut microbiome in pediatric Crohn's Disease. Cell Host Microbe. (2015) 18:489–500. 10.1016/j.chom.2015.09.008 26468751 PMC4633303

[28] Shivappa N Steck SE Hurley TG Hussey JR Hébert JR. Designing and developing a literature-derived, population-based dietary inflammatory index. Public Health Nutr. (2014) 17:1689–96. 10.1017/S1368980013002115 23941862 PMC3925198

[29] Cavicchia PP Steck SE Hurley TG Hussey JR Ma Y Ockene IS. A new dietary inflammatory index predicts interval changes in serum high-sensitivity C-reactive protein. J Nutr. (2009) 139:2365–72. 10.3945/jn.109.114025 19864399 PMC2777480

[30] Shivappa N Wirth MD Murphy EA Hurley TG Hébert JR. Association between the Dietary Inflammatory Index (DII) and urinary enterolignans and C-reactive protein from the National Health and Nutrition Examination Survey-2003-2008. Eur J Nutr. (2019) 58:797–805. 10.1007/s00394-018-1690-5 29675557

[31] Albenberg LG Wu GD. Diet and the intestinal microbiome: associations, functions, and implications for health and disease. Gastroenterology. (2014) 146:1564–72. 10.1053/j.gastro.2014.01.058 24503132 PMC4216184

[32] Kedia S Rampal R Paul J Ahuja V. Gut microbiome diversity in acute infective and chronic inflammatory gastrointestinal diseases in North India. J Gastroenterol. (2016) 51:660–71. 10.1007/s00535-016-1193-1 26994772

[33] Mitsou EK Kakali A Antonopoulou S Mountzouris KC Yannakoulia M Panagiotakos DB. Adherence to the mediterranean diet is associated with the gut microbiota pattern and gastrointestinal characteristics in an adult population. Br J Nutr. (2017) 117:1645–55. 10.1017/S0007114517001593 28789729

[34] De Filippis F Pellegrini N Vannini L Jeffery IB La Storia A Laghi L. High-level adherence to a Mediterranean diet beneficially impacts the gut microbiota and associated metabolome. Gut. (2016) 65:1812–21. 10.1136/gutjnl-2015-309957 26416813

[35] Agustí A García-Pardo MP López-Almela I Campillo I Maes M Romaní-Pérez M. Interplay between the gut-brain axis, obesity and cognitive function. Front Neurosci. (2018) 12:155. 10.3389/fnins.2018.00155 29615850 PMC5864897

[36] Liu XX Jiao B Liao XX Guo LN Yuan ZH Wang X. Analysis of Salivary Microbiome in Patients with Alzheimer's Disease. J Alzheimers Dis. (2019) 72:633–40. 10.3233/JAD-190587 31594229

[37] Riviere GR Riviere KH Smith KS. Molecular and immunological evidence of oral Treponema in the human brain and their association with Alzheimer's disease. Oral Microbiol Immunol. (2002) 17:113–8. 10.1046/j.0902-0055.2001.00100.x 11929559

[38] Dominy SS Lynch C Ermini F Benedyk M Marczyk A Konradi A. Porphyromonas gingivalis in Alzheimer's disease brains: Evidence for disease causation and treatment with small-molecule inhibitors. Sci Adv. (2019) 5:eaau3333. 10.1126/sciadv.aau3333 30746447 PMC6357742

[39] Wu S Liu X Jiang R Yan X Ling Z. Roles and mechanisms of gut microbiota in patients with Alzheimer's Disease. Front Aging Neurosci. (2021) 13:650047. 10.3389/fnagi.2021.650047 34122039 PMC8193064

[40] Sato K Takahashi N Kato T Matsuda Y Yokoji M Yamada M. Aggravation of collagen-induced arthritis by orally administered Porphyromonas gingivalis through modulation of the gut microbiota and gut immune system. Sci Rep. (2017) 7:6955. 10.1038/s41598-017-07196-7 28761156 PMC5537233

[41] Shoemark DK Allen SJ. The microbiome and disease: reviewing the links between the oral microbiome, aging, and Alzheimer's disease. J Alzheimers Dis. (2015) 43:725–38. 10.3233/JAD-141170 25125469

[42] Sochocka M Donskow-Łysoniewska K Diniz BS Kurpas D Brzozowska E Leszek J. The gut microbiome alterations and inflammation-driven pathogenesis of Alzheimer's Disease-a critical review. Mol Neurobiol. (2019) 56:1841–51. 10.1007/s12035-018-1188-4 29936690 PMC6394610

[43] Tran L Greenwood-Van Meerveld B. Age-associated remodeling of the intestinal epithelial barrier. J Gerontol A Biol Sci Med Sci. (2013) 68:1045–56. 10.1093/gerona/glt106 23873964 PMC3738030

[44] Harding A Gonder U Robinson SJ Crean S Singhrao SK. Exploring the Association between Alzheimer's Disease, oral health, microbial endocrinology and nutrition. Front Aging Neurosci. (2017) 9:398. 10.3389/fnagi.2017.00398 29249963 PMC5717030

[45] Battle DE. Diagnostic and Statistical Manual of Mental disorders (DSM). CoDAS. (2013) 25:191–2. 10.1590/s2317-17822013000200017 24413388

[46] Spering CC Hobson V Lucas JA Menon CV Hall JR O'Bryant SE. Diagnostic accuracy of the MMSE in detecting probable and possible Alzheimer's disease in ethnically diverse highly educated individuals: an analysis of the NACC database. J Gerontol A Biol Sci Med Sci. (2012) 67:890–6. 10.1093/gerona/gls006 22396476 PMC3403860

[47] Zhao WH Huang ZP Zhang X Li HE Willett W Wang JL. Reproducibility and validity of a chinese food frequency questionnaire. Biomed Environ Sci. (2010) S1:38. 10.1016/S0895-3988(11)60014-716672199

[48] Willett W. Nutritional Epidemiology |Issues in Analysis and Presentation of Dietary Data. (2012).

[49] Craig CL Marshall AL Sjöström M Bauman AE Booth ML Ainsworth BE. International physical activity questionnaire: 12-country reliability and validity. Med Sci Sports Exerc. (2003) 35:1381–95. 10.1249/01.MSS.0000078924.61453.FB 12900694

[50] Rademacher SWH Zaura E Kleverlaan CJ Buijs MJ Crielaard W Loos BG. Qualitative and quantitative differences in the subgingival microbiome of the restored and unrestored teeth. J Periodontal Res. (2019) 54:405–12. 10.1111/jre.12642 30734922 PMC6766957

[51] Segata N Izard J Waldron L Gevers D Miropolsky L Garrett WS. Metagenomic biomarker discovery and explanation. Genome Biol. (2011) 12:R60. 10.1186/gb-2011-12-6-r60 21702898 PMC3218848

[52] Willett WC Howe GR Kushi LH. Adjustment for total energy intake in epidemiologic studies. Am J Clin Nutr. (1997) 65(4 Suppl):1220S-8S; discussion 9S-31S. 10.1093/ajcn/65.4.1220S 9094926

[53] Parks DH Tyson GW Hugenholtz P Beiko RG. STAMP statistical analysis of taxonomic and functional profiles. Bioinformatics. (2014) 30:3123–4. 10.1093/bioinformatics/btu494 25061070 PMC4609014

[54] Shivappa N Hebert JR Rietzschel ER De Buyzere ML Langlois M Debruyne E. Associations between dietary inflammatory index and inflammatory markers in the Asklepios Study. Br J Nutr. (2015) 113:665–71. 10.1017/S000711451400395X 25639781 PMC4355619

[55] Julia C Assmann KE Shivappa N Hebert JR Wirth MD Hercberg S. Long-term associations between inflammatory dietary scores in relation to long-term C-reactive protein status measured 12 years later: findings from the Supplémentation en Vitamines et Minéraux Antioxydants (SU. VIMAX) cohort. Br J Nutr. (2017) 117:306–14. 10.1017/S0007114517000034 28166841

[56] Hodge AM Bassett JK Shivappa N Hébert JR English DR Giles GG. Dietary inflammatory index, Mediterranean diet score, and lung cancer: a prospective study. Cancer Causes Control. (2016) 27:907–17. 10.1007/s10552-016-0770-1 27294725 PMC5550291

[57] Casas R Sacanella E Urpí-Sardà M Corella D Castañer O Lamuela-Raventos RM. Long-term immunomodulatory effects of a mediterranean diet in adults at high risk of cardiovascular disease in the PREvención con DIeta MEDiterránea (PREDIMED) Randomized Controlled Trial. J Nutr. (2016) 146:1684–93. 10.3945/jn.115.229476 27440261

[58] Casas R Urpi-Sardà M Sacanella E Arranz S Corella D Castañer O. Anti-Inflammatory effects of the mediterranean diet in the early and late stages of atheroma plaque development. Mediators Inflamm. (2017) 2017:3674390. 10.1155/2017/3674390 28484308 PMC5412172

[59] Motta M Imbesi R Di Rosa M Stivala F Malaguarnera L. Altered plasma cytokine levels in Alzheimer's disease: correlation with the disease progression. Immunol Lett. (2007) 114:46–51. 10.1016/j.imlet.2007.09.002 17949824

[60] King E O'Brien JT Donaghy P Morris C Barnett N Olsen K. Peripheral inflammation in prodromal Alzheimer's and Lewy body dementias. J Neurol Neurosurg Psychiatry. (2018) 89:339–45. 10.1136/jnnp-2017-317134 29248892 PMC5869446

[61] Brodie C Goldreich N Haiman T Kazimirsky G. Functional IL-4 receptors on mouse astrocytes: IL-4 inhibits astrocyte activation and induces NGF secretion. J Neuroimmunol. (1998) 81:20–30. 10.1016/S0165-5728(97)00154-9 9521602

[62] Suh HS Zhao ML Derico L Choi N Lee SC. Insulin-like growth factor 1 and 2 (IGF1, IGF2) expression in human microglia: differential regulation by inflammatory mediators. J Neuroinflammation. (2013) 10:37. 10.1186/1742-2094-10-37 23497056 PMC3607995

[63] Anderson AC Rothballer M Altenburger MJ Woelber JP Karygianni L Vach K. Long-term fluctuation of oral biofilm microbiota following different dietary phases. Appl Environ Microbiol. (2020) 86:e01421–20. 10.1128/AEM.01421-20 32801176 PMC7531951

[64] Millen AE Dahhan R Freudenheim JL Hovey KM Li L McSkimming DI. Dietary carbohydrate intake is associated with the subgingival plaque oral microbiome abundance and diversity in a cohort of postmenopausal women. Sci Rep. (2022) 12:2643. 10.1038/s41598-022-06421-2 35173205 PMC8850494

[65] Jockel-Schneider Y Schlagenhauf U Stölzel P Goßner S Carle R Ehmke B. Nitrate-rich diet alters the composition of the oral microbiota in periodontal recall patients. J Periodontol. (2021) 92:1536–45. 10.1002/JPER.20-0778 33742692

[66] Zheng J Hoffman KL Chen JS Shivappa N Sood A Browman GJ. Dietary inflammatory potential in relation to the gut microbiome: results from a cross-sectional study. Br J Nutr. (2020) 124:931–42. 10.1017/S0007114520001853 32475373 PMC7554089

[67] Tian Z Zhuang X Zhuo S Zhu Y Hu S Zhao M. Dietary inflammatory potential mediated gut microbiota and metabolite alterations in Crohn's disease: A fire-new perspective. Clin Nutr. (2022) 41:1260–71. 10.1016/j.clnu.2022.04.014 35504169

[68] De Filippo C Cavalieri D Di Paola M Ramazzotti M Poullet JB Massart S. Impact of diet in shaping gut microbiota revealed by a comparative study in children from Europe and rural Africa. Proc Natl Acad Sci U S A. (2010) 107:14691–6. 10.1073/pnas.1005963107 20679230 PMC2930426

[69] Matijašić BB Obermajer T Lipoglavšek L Grabnar I Avguštin G Rogelj I. Association of dietary type with fecal microbiota in vegetarians and omnivores in Slovenia. Eur J Nutr. (2014) 53:1051–64. 10.1007/s00394-013-0607-6 24173964

[70] Segata N Haake SK Mannon P Lemon KP Waldron L Gevers D. Composition of the adult digestive tract bacterial microbiome based on seven mouth surfaces, tonsils, throat and stool samples. Genome Biol. (2012) 13:R42. 10.1186/gb-2012-13-6-r42 22698087 PMC3446314

[71] Flemer B Warren RD Barrett MP Cisek K Das A Jeffery IB. The oral microbiota in colorectal cancer is distinctive and predictive. Gut. (2018) 67:1454–63. 10.1136/gutjnl-2017-314814 28988196 PMC6204958

[72] Kong C Gao R Yan X Huang L Qin H. Probiotics improve gut microbiota dysbiosis in obese mice fed a high-fat or high-sucrose diet. Nutrition. (2019) 60:175–84. 10.1016/j.nut.2018.10.002 30611080

[73] Kovatcheva-Datchary P Nilsson A Akrami R Lee YS De Vadder F Arora T. Dietary fiber-induced improvement in glucose metabolism is associated with increased abundance of prevotella. Cell Metab. (2015) 22:971–82. 10.1016/j.cmet.2015.10.001 26552345

[74] An R Liu G Khan N Yan H Wang Y. Dietary habits and cognitive impairment risk among oldest-old Chinese. J Gerontol B Psychol Sci Soc Sci. (2019) 74:474–83. 10.1093/geronb/gbw170 28184889

[75] Zhou Y Wang Y Quan M Zhao H Jia J. Gut microbiota changes and their correlation with cognitive and neuropsychiatric symptoms in Alzheimer's Disease. J Alzheimers Dis. (2021) 81:583–95. 10.3233/JAD-201497 33814442

[76] Zhang X Wang Y Liu W Wang T Wang L Hao L. Diet quality, gut microbiota, and microRNAs associated with mild cognitive impairment in middle-aged and elderly Chinese population. Am J Clin Nutr. (2021) 114:429–40. 10.1093/ajcn/nqab078 33871591

[77] Louis P Hold GL Flint HJ. The gut microbiota, bacterial metabolites and colorectal cancer. Nat Rev Microbiol. (2014) 12:661–72. 10.1038/nrmicro3344 25198138

[78] Koh A De Vadder F Kovatcheva-Datchary P Bäckhed F. From dietary fiber to host physiology: short-chain fatty acids as key bacterial metabolites. Cell. (2016) 165:1332–45. 10.1016/j.cell.2016.05.041 27259147

[79] Jiang C Li G Huang P Liu Z Zhao B. The gut microbiota and Alzheimer's Disease. J Alzheimers Dis. (2017) 58:1–15. 10.3233/JAD-161141 28372330

[80] Jeffery IB Toole PW. Diet-microbiota interactions and their implications for healthy living. Nutrients. (2013) 5:234–52 10.3390/nu5010234 23344252 PMC3571646

[81] Chen X Maguire B Brodaty H O'Leary F. Dietary patterns and cognitive health in older adults: a systematic review. J Alzheimers Dis. (2019) 67:583–619. 10.3233/JAD-180468 30689586

[82] Rajaram S Jones J Lee GJ. Plant-based dietary patterns, plant foods, and age-related cognitive decline. Adv Nutr. (2019) 10:S422–s36. 10.1093/advances/nmz081 31728502 PMC6855948

[83] Cattaneo A Cattane N Galluzzi S Provasi S Lopizzo N Festari C. Association of brain amyloidosis with pro-inflammatory gut bacterial taxa and peripheral inflammation markers in cognitively impaired elderly. Neurobiol Aging. (2017) 49:60–8. 10.1016/j.neurobiolaging.2016.08.019 27776263

[84] Van Eldik LJ Carrillo MC Cole PE Feuerbach D Greenberg BD Hendrix JA. The roles of inflammation and immune mechanisms in Alzheimer's disease. Alzheimers Dement. (2016) 2:99–109. 10.1016/j.trci.2016.05.001 29067297 PMC5644267

[85] McDaniel J McDaniel S Samiano BJ Marrujo M Kingsley K Howard KM. Microbial screening reveals oral site-specific locations of the periodontal pathogen selenomonas noxia. Curr Issues Mol Biol. (2021) 43:353–64. 10.3390/cimb43010029 34204609 PMC8929098

[86] Kolenbrander PE Andersen RN Moore LV. Coaggregation of fusobacterium nucleatum, selenomonas flueggei, selenomonas infelix, selenomonas noxia, and selenomonas sputigena with strains from 11 genera of oral bacteria. Infect Immun. (1989) 57:3194–203. 10.1128/iai.57.10.3194-3203.1989 2777378 PMC260789

[87] Tanner A Maiden MF Macuch PJ Murray LL Kent RL Jr. Microbiota of health, gingivitis, and initial periodontitis. J Clin Periodontol. (1998) 25:85–98. 10.1111/j.1600-051X.1998.tb02414.x 9495607

[88] Socransky SS Haffajee AD Cugini MA Smith C Kent RL Jr. Microbial complexes in subgingival plaque. J Clin Periodontol. (1998) 25:134–44. 10.1111/j.1600-051X.1998.tb02419.x 9495612

[89] Bui TPN de Vos WM Plugge CM. Anaerostipes rhamnosivorans sp. nov., a human intestinal, butyrate-forming bacterium. Int J Syst Evol Microbiol. (2014) 64(Pt 3):787–93. 10.1099/ijs.0.055061-0 24215821

[90] Lee JY Kang W Shin NR Hyun DW Kim PS Kim HS. Anaerostipes hominis sp. nov., a novel butyrate-producing bacteria isolated from faeces of a patient with Crohn's disease. Int J Syst Evol Microbiol. (2021) 71:12. 10.1099/ijsem.0.005129 34870576

[91] Teixeira CG Fusieger A Milião GL Martins E Drider D Nero LA. Weissella: an emerging bacterium with promising health benefits. Probiotics Antimicrob Proteins. (2021) 13:915–25. 10.1007/s12602-021-09751-1 33565028

[92] Kim JW Jung BH Lee JH Yoo KY Lee H Kang MS. Effect of Weissella cibaria on the reduction of periodontal tissue destruction in mice. J Periodontol. (2020) 91:1367–74. 10.1002/JPER.19-0288 32017095

[93] Hou M Xu G Ran M Luo W Wang H. APOE-ε4 carrier status and gut microbiota dysbiosis in patients with Alzheimer Disease. Front Neurosci. (2021) 15:619051. 10.3389/fnins.2021.619051 33732104 PMC7959830

[94] Ho L Ono K Tsuji M Mazzola P Singh R Pasinetti GM. Protective roles of intestinal microbiota derived short chain fatty acids in Alzheimer's disease-type beta-amyloid neuropathological mechanisms. Expert Rev Neurother. (2018) 18:83–90. 10.1080/14737175.2018.1400909 29095058 PMC5958896

[95] Qian Y Yang X Xu S Wu C Song Y Qin N. Alteration of the fecal microbiota in Chinese patients with Parkinson's disease. Brain Behav Immun. (2018) 70:194–202. 10.1016/j.bbi.2018.02.016 29501802

[96] Shah MM Odoyo E Ichinose Y. Epidemiology and pathogenesis of providencia alcalifaciens infections. Am J Trop Med Hyg. (2019) 101:290–3. 10.4269/ajtmh.18-0376 31218997 PMC6685554

[97] Kurmasheva N Vorobiev V Sharipova M Efremova T Mardanova A. The potential virulence factors of providencia stuartii: motility, adherence, and invasion. Biomed Res Int. (2018) 2018:3589135. 10.1155/2018/3589135 29682537 PMC5841065

[98] Smith CJ Emge JR Berzins K Lung L Khamishon R Shah P. Probiotics normalize the gut-brain-microbiota axis in immunodeficient mice. Am J Physiol Gastrointest Liver Physiol. (2014) 307:G793–802. 10.1152/ajpgi.00238.2014 25190473 PMC4200314

[99] Emge JR Huynh K Miller EN Kaur M Reardon C Barrett KE. Modulation of the microbiota-gut-brain axis by probiotics in a murine model of inflammatory bowel disease. Am J Physiol Gastrointest Liver Physiol. (2016) 310:G989–98. 10.1152/ajpgi.00086.2016 27056723

[100] Akbari E Asemi Z Daneshvar Kakhaki R Bahmani F Kouchaki E Tamtaji OR. Effect of probiotic supplementation on cognitive function and metabolic status in Alzheimer's Disease: a randomized, double-blind and controlled trial. Front Aging Neurosci. (2016) 8:256. 10.3389/fnagi.2016.00256 27891089 PMC5105117

[101] Graves DT Corrêa JD Silva TA. The oral microbiota is modified by systemic diseases. J Dent Res. (2019) 98:148–56. 10.1177/0022034518805739 30359170 PMC6761737

[102] Corrêa JD Calderaro DC Ferreira GA Mendonça SM Fernandes GR Xiao E. Subgingival microbiota dysbiosis in systemic lupus erythematosus: association with periodontal status. Microbiome. (2017) 5:34. 10.1186/s40168-017-0252-z 28320468 PMC5359961

[103] Xiao E Mattos M Vieira GHA Chen S Corrêa JD Wu Y. Diabetes Enhances IL-17 Expression and Alters the Oral Microbiome to Increase Its Pathogenicity. Cell Host Microbe. (2017) 22:120–8.e4. 10.1016/j.chom.2017.06.014 28704648 PMC5701758

