# Supplementary material for: Different oral and gut microbial profiles in those with Alzheimer's disease consuming anti-inflammatory diets
Source: Front Nutr. 2022 Sep 15;9:974694. doi: 10.3389/fnut.2022.974694 (PMC9521405; doi:10.3389/fnut.2022.974694)
Supplement: Supplementary file 1 [file Data_Sheet_1.pdf]

# *Supplementary Material*

## Association of different DII levels with inflammatory markers in AD patients

Multiple linear regression was used to analyze inflammatory markers among three groups of DII patients with AD, with model adjustment for confounders. In Model 1, we did not adjust for covariates. In Model 2, we adjusted for age and gender. In Model 3, we adjusted for the variables in Model 2 with the addition of education, BMI, smoking, alcohol consumption, physical activity, hypertension, diabetes, hyperlipidaemia, coronary heart disease and cerebrovascular disease. Tests for trends were performed by assigning the median value for each tertile and modeling this as a continuous variable. And the results showed that none of the inflammatory markers were statistically significant (p > 0.05).

**Supplementary Table 1. The multiple linear regression analysis of different DII levels and inflammatory indicators in AD patients**

| **inflammatory indicators** | **DII tertiles** | **Unadjusted** | | **Model 1** | | **Model 2** | |
|---|---|---|---|---|---|---|---|
| | | **β（95%CI）** | ***p*-trend** | **β（95%CI）** | ***p*-trend** | **β（95%CI）** | ***p*-trend** |
| IL-1β | | | 0.850 | | 0.717 | | 0.730 |
| | T1 | reference | | reference | | reference | |
| | T2 | -0.03(-0.48-0.43) | | 0.01(-0.46-0.48) | | 0.10(-0.50-0.69) | |
| | T3 | 0.05(-0.40-0.51) | | 0.09(-0.38-0.57) | | 0.09(-0.55-0.73) | |
| IL-4 | | | 0.664 | | 0.739 | | 0.986 |
| | T1 | reference | | reference | | reference | |
| | T2 | -0.13(-0.52-0.25) | | -0.13(-0.53-0.27) | | -0.31(-0.83-0.22) | |
| | T3 | 0.12(-0.27-0.50) | | 0.10(-0.31-0.50) | | 0.12(-0.45-0.68) | |
| IL-6 | | | 0.863 | | 0.709 | | 0.650 |
| | T1 | reference | | reference | | reference | |
| | T2 | 0.02(-0.36-0.40) | | 0.09(-0.28-0.47) | | 0.03(-0.46-0.51) | |
| | T3 | -0.04(-0.42-0.34) | | 0.06(-0.32-0.44) | | 0.13(-0.39-0.65) | |
| IL-10 | | | 0.854 | | 0.874 | | 0.173 |
| | T1 | reference | | reference | | reference | |
| | T2 | 0.14(-0.43-0.70) | | 0.18(-0.40-0.76) | | -0.31(-0.92-0.30) | |
| | T3 | -0.08(-0.65-0.48) | | -0.08(-0.67-0.50) | | -0.41(-1.07-0.24) | |
| IL-12 | | | 0.149 | | 0.217 | | |
| | T1 | reference | | reference | | reference | |
| | T2 | -0.40(-0.98-0.18) | | -0.38(-0.98-0.22) | | -0.33(-1.14-0.48) | |
| | T3 | | | | | | |

| inflammatory indicators | DII tertiles | Unadjusted | | Model 1 | | Model 2 | |
|---|---|---|---|---|---|---|---|
| | | β（95%CI） | *p*-trend | β（95%CI） | *p*-trend | β（95%CI） | *p*-trend |
| | | -0.39(-0.97-0.19) | | -0.35(-0.95-0.26) | | 0.22(-0.64-1.09) | |
| TNF-α | | | 0.202 | | 0.383 | | 0.448 |
| | T1 | reference | | reference | | reference | |
| | T2 | -0.31(-0.64-0.02) | | -0.28(-0.62-0.05) | | -0.10(-0.44-0.25) | |
| | T3 | -0.18(-0.51-0.15) | | -0.12(-0.46-0.22) | | -0.13(-0.50-0.24) | |
| hs-CRP | | | 0.157 | | 0.251 | | 0.126 |
| | T1 | reference | | reference | | reference | |
| | T2 | -0.02(-0.39-0.34) | | 0.02(-0.35-0.40) | | 0.05(-0.42-0.51) | |
| | T3 | -0.28(-0.65-0.08) | | -0.25(-0.63-0.13) | | -0.48(-0.99-0.02) | |
| C3 | | | 0.958 | | 0.999 | | 0.623 |
| | T1 | reference | | reference | | reference | |
| | T2 | -0.15(-0.62-0.32) | | -0.15(-0.64-0.33) | | -0.09(-0.68-0.50) | |
| | T3 | 0.04(-0.43-0.51) | | 0.03(-0.47-0.52) | | -0.15(-0.78-0.49) | |

Model1: unadjusted; Model 2: adjusted for age and sex; Model 3: adjusted for education, BMI, smoking, alcohol consumption, physical activity, hypertension, diabetes, hyperlipidaemia, coronary heart disease, and cerebrovascular disease.

**Crucial bacteria responsible for differentiating the most anti-inflammatory diet group from the most pro-inflammatory diet group**

To further explore the all alterations of oral and gut microbiomes between anti-inflammatory diet group and pro-inflammatory diet group, we used LEfSe (LDA score cut off > 2.0) analysis to identify the key taxa responsible for the differences in the compositions of the oral and gut microbiota. However, there were no significant differences among three groups in AD patients (Supplementary Figure 1).

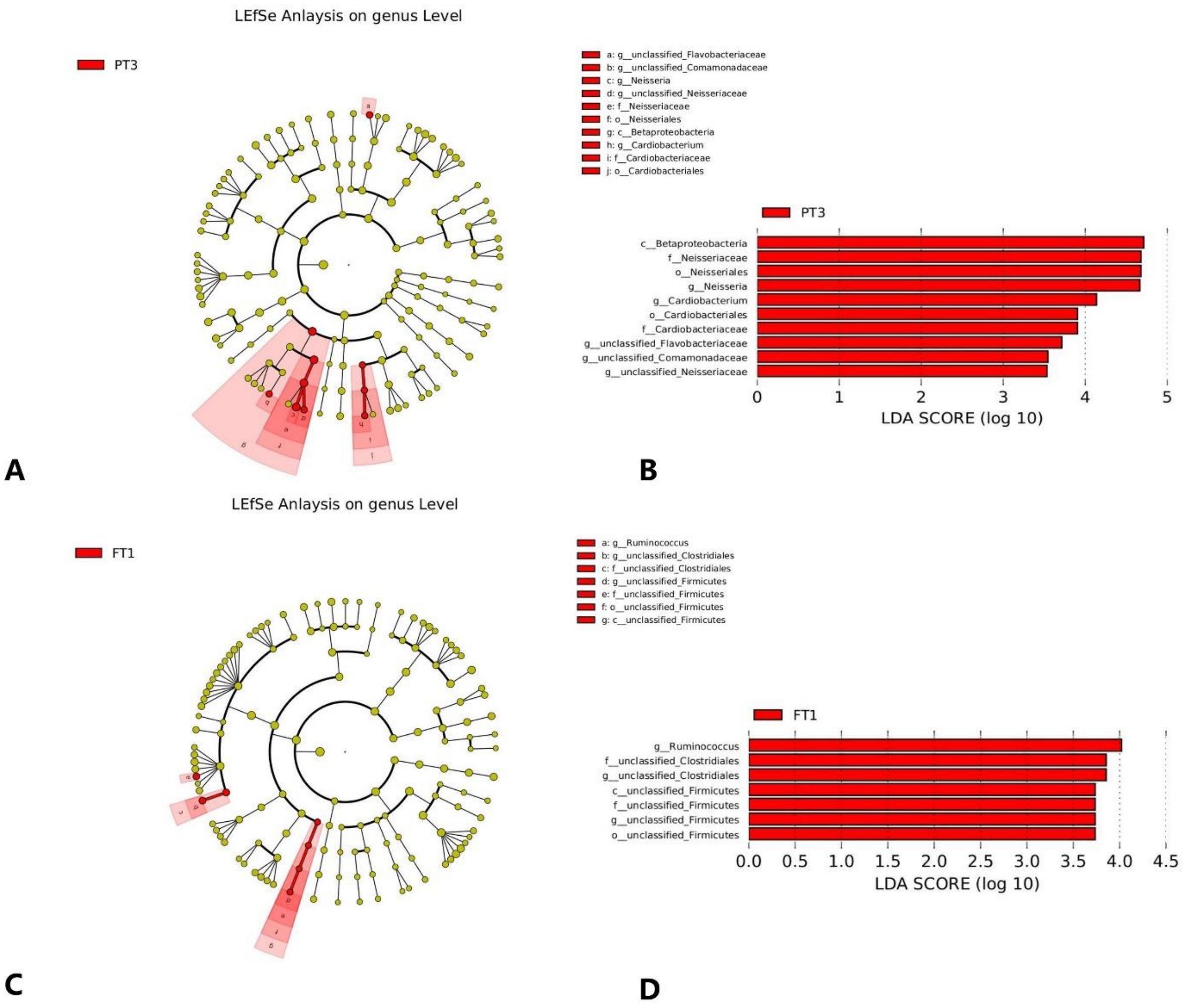


**Supplementary Figure 1.** Key taxonomic differences of oral and gut microbiota in three groups with AD patients. Cladogram using LEfSe method indicated the phylogenetic distribution of the oral (A-B) and gut (C-D) microbiota. Each circle's diameter was proportional to the taxon's abundance.
